# Electrochemical Carbon Dioxide Reduction to Methanol on Copper‐Based Catalysts: Mechanistic Insights and Industrial Prospects

**DOI:** 10.1002/adma.202514994

**Published:** 2026-01-07

**Authors:** Debabrata Bagchi, Carsten Walter, Venkata S. R. K. Tandava, Yasmin Lucero Cobos‐Becerra, Jack C. Q. Fletcher, Nico Fischer, Tobias Sontheimer, Prashanth W. Menezes

**Affiliations:** ^1^ Department of Materials Chemistry for Catalysis Helmholtz‐Zentrum Berlin für Materialien und Energie Berlin Germany; ^2^ Strategy Department of Energy and Information Helmholtz‐Zentrum Berlin für Materialien und Energie Berlin Germany; ^3^ Catalysis Institute Department of Chemical Engineering University of Cape Town Rondebosch Cape Town South Africa; ^4^ Department of Chemistry Technical University of Berlin Berlin Germany; ^5^ Centre for Future Materials University of Southern Queensland Toowoomba Queensland Australia

**Keywords:** active sites, CO_2_ to methanol, in situ/operando investigations, reaction mechanism, techno‐economics

## Abstract

Electrochemical CO_2_ reduction (ECO_2_R) offers a promising route to convert CO_2_ into high‐value‐added chemicals using renewable energy. Among the diverse ECO_2_R products, the selective conversion of CO_2_ to methanol (CH_3_OH) holds significant industrial importance as a fuel and chemical feedstock. This review provides a comprehensive overview of recent progress in Copper (Cu)‐based catalysts for selective ECO_2_R to CH_3_OH. Key advancements in catalyst design and synthesis are discussed, followed by mechanistic insights obtained through computational modeling and advanced characterization techniques. Special focus is given to the structure‐activity relationship that controls CH_3_OH selectivity, disclosing the importance of intermediate stabilization and electronic structure tuning. Further, state‐of‐the‐art Cu‐based materials and benchmarking their performances under various operating conditions, including the role of electrolyzer configurations, electrolytes, and ion‐exchange membranes, is summarized. Moreover, we analyze challenges in upscaling, such as stability, selectivity under high current densities, and integration with renewable energy sources. Besides, the potential of tandem and hybrid systems to improve reaction pathways is also emphasized. Finally, techno‐economic considerations are explored to evaluate the feasibility of large‐scale CH_3_OH production. By combining fundamental understanding with practical implementation, this review provides strategic direction toward the rational design of Cu‐based electrocatalysts and the development of commercially viable ECO_2_R systems for sustainable CH_3_OH synthesis.

## Graphical abstract

This review presents a focused and integrated perspective on copper‐based catalysts for the selective electrochemical reduction of CO_2_ to methanol. It elucidates active site dynamics, mechanistic pathways, and structure–activity relationships, while connecting fundamental insights with catalyst design, reactor engineering, and techno‐economic considerations to guide the development of commercially viable, renewable‐energy‐driven methanol production technologies.

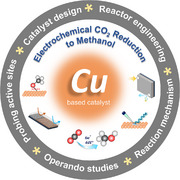

## 1 Introduction

**FIGURE 1:**
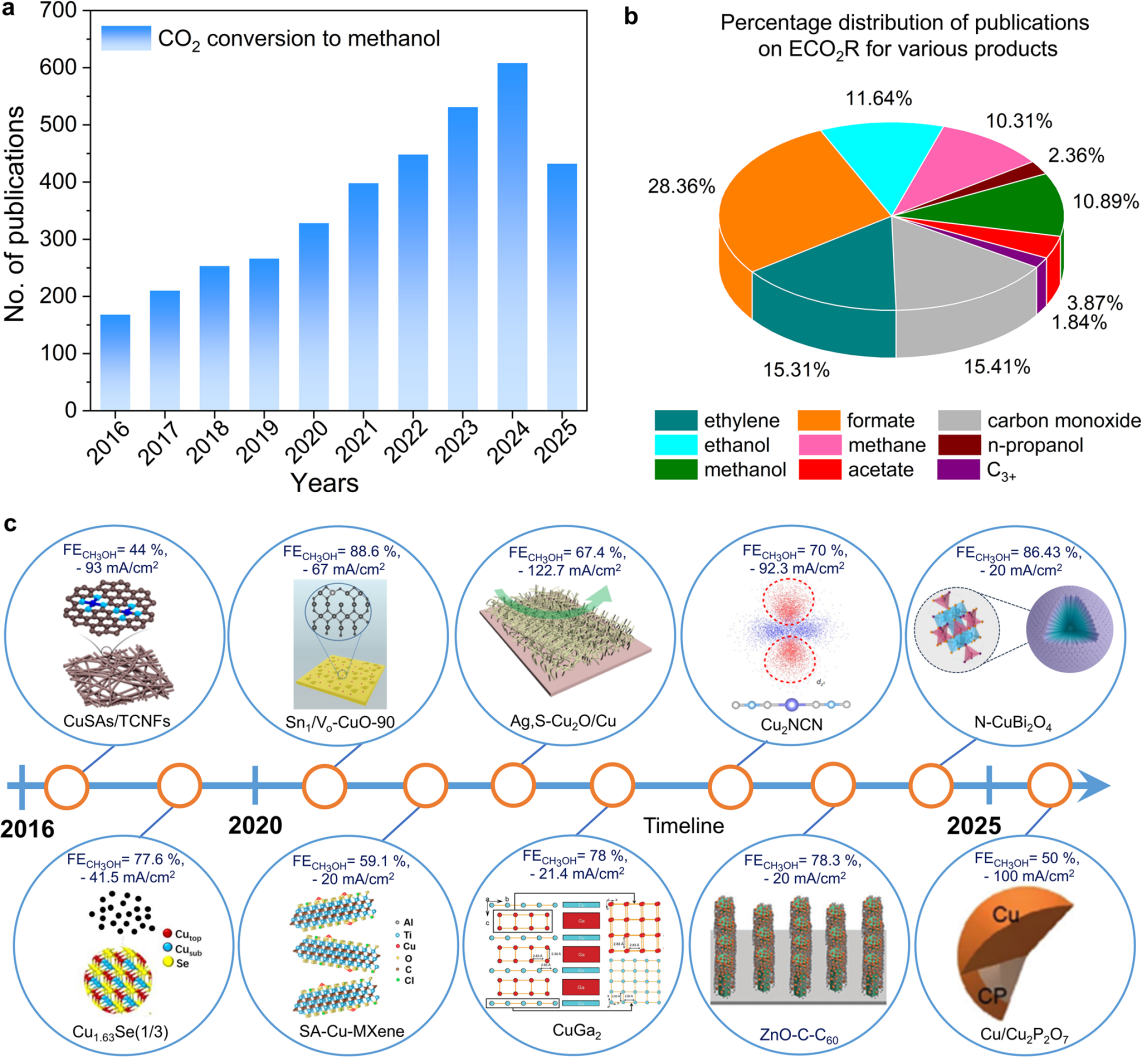
(a) Number of research papers published in the last 10 years (2016–2025) on selective conversion of CO_2_ into CH_3_OH. (b) Illustration of the percentage distribution of around 4000 publications during 2021–2025 on ECO_2_R, focusing on the selective formation of various products. Data obtained from the Web of Science Core Collection database. (c) Representative timeline illustrating the evolution of Cu ‐based catalysts for selective methanol production via ECO_2_R. The figure highlights key advancements in catalyst design strategies from copper oxide, selenide, single atom to heterostructure, demonstrating the improvement in current density, selectivity, and mechanistic understanding toward methanol formation [31, 32, 33, 34, 35, 36, 37, 38, 39, 40]. Image for CuSAs/TCNFs: Reproduced with permission [34]. Copyright 2019, American Chemical Society. Image for Cu_1.63_Se(1/3): Reproduced with permission [35]. Copyright 2019, Springer Nature. Image for Sn_1_/V_o_‐CuO‐90: Reproduced with permission [38]. Copyright 2021, Wiley‐VCH. Image for SA‐Cu‐MXene: Reproduced with permission [33]. Copyright 2021, American Chemical Society. Image for Ag,S‐Cu_2_O/Cu: Reproduced with permission [37]. Copyright 2022, Springer Nature. Image for CuGa_2_: Reproduced with permission [36]. Copyright 2022, Wiley‐VCH. Image for Cu_2_NCN: Reproduced with permission [32]. Copyright 2023, Springer Nature. Image for ZnO‐C‐C_60_: Reproduced with permission [39]. Copyright 2023, Wiley‐VCH. Image for N‐CuBi_2_O_4_: Reproduced with permission [40]. Copyright 2024, Elsevier. Image for Cu/Cu_2_P_2_O_7_: Reproduced with permission [31]. Copyright 2025, Wiley‐VCH.

Over the past decades, industrial activities strongly dependent on fossil energy sources have significantly impacted the Earth's atmosphere, leading to a sharp increase in the concentration of the greenhouse gas carbon dioxide (CO_2_), thereby accelerating climate change and global warming [1, 2]. To mitigate these effects and meet the rising energy demand, hydrogen (H_2_) production through technologies such as water electrolyzer cells (WEC), i.e., polymer electrolyte membrane (PEM), alkaline electrolyzer cells (AEC), membrane electrode assembly (MEA), and solid oxide electrolysis (SOEL) has emerged as a promising approach for clean fuel generation and green energy storage [3, 4, 5, 6, 7, 8]. However, with atmospheric CO_2_ levels continuing to rise at an alarming rate, other technologies aimed at decarbonization, carbon capture and storage, as well as carbon recycling are receiving increasing attention [1, 9, 10]. Among them, numerous carbon recycling technologies, particularly the electrochemical CO_2_ reduction (ECO_2_R) reaction combined with renewable energy sources, present a sustainable and green approach to both lowering atmospheric CO_2_ levels and converting CO_2_ into high‐value chemicals and fuels [11, 12, 13]. Despite substantial progress over the last three decades, no catalyst has yet achieved the ideal combination of cost‐efficiency, activity, selectivity, and long‐term stability required for large‐scale ECO_2_R applications [14]. The growing interest in CO_2_ to CH_3_OH conversion is revealed by a consistent increment in related publications over the past decade (Figure 1a).

In the field of ECO_2_R, a wide range of materials under various reaction conditions have been extensively explored to catalyze CO_2_ into industrially important products such as CH_3_OH, carbon monoxide (CO), formate (HCOO^−^), acetate (CH_3_COO^−^), methane (CH_4_), ethylene (C_2_H_4_), ethanol (CH_3_CH_2_OH), and propanol (CH_3_CH_2_CH_2_OH) (Figure 1b) [15]. In particular, the ECO_2_R to CH_3_OH is of expanding importance in both chemical manufacturing and energy applications [10, 15]. Methanol, as a key chemical feedstock for the manufacturing of formaldehyde, acetic acid, olefins, silicone and plastics, had an estimated global CH_3_OH demand of 110 million metric tons (MMT) in 2023 alone [16, 17]. This demand is projected to exceed 170 MMT by 2050 and could even reach 500 MMT if it significantly replaces fossil fuels as a fuel substitute in the marine and aviation sectors [17, 18]. Its volumetric energy density (16 MJ L^−1^) and H_2_ density (≈100 g H_2_ L^−1^) surpass that of formic acid and compressed or liquefied H_2_, in practical storage applications. With the techno‐economic outlook to be competitive with fossil‐based syngas routes, ECO_2_R to CH_3_OH is uniquely suited for energy storage, transportation, and stationary power generation [17, 19, 20]. Although key challenges remain, particularly in achieving high selectivity and long‐term stability at industrially relevant conditions, various catalysts such as CoPc [21], Co‐corrole [22], FeTEsCCl [16], Pd/SnO_2_ [16], and RuO_2_/TiO_2_ show promise [23]. Among the various catalyst candidates, Cu remains a significant attention due to the ideal binding energy of important intermediates such as *CO and *COH and its ability to suppress competing reactions such as HER [24, 25, 26, 27, 28, 29]. Over the past decades, systematic advancements in the structural and electronic engineering of Cu‐based catalysts have obtained critical correlations between surface coordination environments, oxidation states, and intermediate stabilization pathways, thereby enabling a progressive improvement in methanol selectivity and its mechanistic understanding during ECO_2_R (Figure 1c).

**FIGURE 2:**
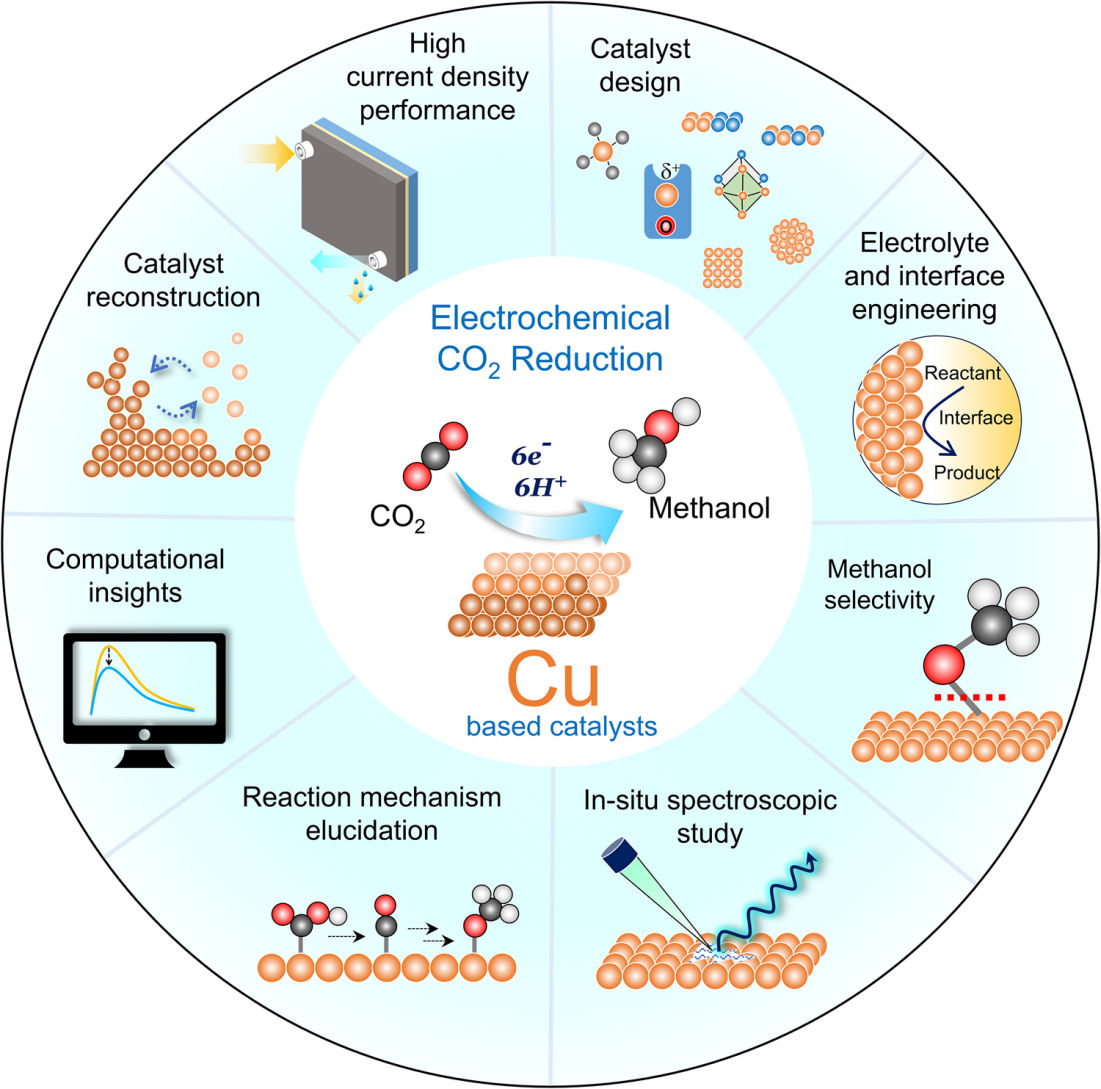
Schematic representation of the central theme of this review, highlighting the dynamic structural and chemical evolution of Cu‐based catalysts, and the strategic integration of in situ diagnostic techniques to elucidate reaction mechanisms, active site transformations, and selectivity pathways ECO_2_R to CH_3_OH.

In this review, we focus on the most recent advances in ECO_2_R to CH_3_OH with Cu‐based materials (Figure 2). We begin by reviewing the electrocatalytic performance of numerous Cu‐based compounds tested for ECO_2_R in neutral, alkaline, and acidic media, with attention to their design strategies and electrocatalytic performance. Following, to provide a fundamental understanding of ECO_2_R, we discuss mechanistic insights, reaction pathways, competing side reactions, key intermediates, and the effect of the local reaction environment on the selective formation of CH_3_OH. Special emphasis has been given to the understanding of the nature of precatalysts and active sites in Cu‐based electrocatalysts, their structure‐activity relation under reaction conditions, surface/bulk modifications, and electronic configurations by discussing the insights derived from in situ and operando analysis. We also examine electrocatalytic performance metrics and benchmarking protocols, as well as evaluation parameters, state‐of‐the‐art catalysts, and the influence of reactor design and operating conditions. Finally, we outline future perspectives and directions of Cu‐based ECO_2_R catalysts aimed at advancing CH_3_OH production. This includes promising design strategies by employing advanced reactor engineering, up‐scaling potential, commercialization challenges, utilization of tandem electrolysis approach, integration of CO_2_ capture and renewable energy sources, and key areas for fundamental research. Unlike broader reviews covering various catalyst systems or products, this work specifically provides a focused and integrated perspective on Cu‐based electrocatalysts for CH_3_OH production, linking mechanistic insights with system‐level considerations to bridge fundamental understanding and practical application [1, 10, 11, 14, 30]. We believe that this review will provide deep insights into the field of Cu‐based ECO_2_R to CH_3_OH and will serve as a valuable resource and inspiration for future research.

The activity, selectivity, and overall performance of ECO_2_R are mainly governed by the electrocatalyst material. In the previous sections of the review, the literature overview of the catalysts was discussed extensively, and below we present the state‐of‐the‐art reported Cu‐based catalysts with detailed experimental conditions and observed electrochemical performance reported over the years.

## 2 Copper as a Catalyst for Methanol Formation

Cu exhibits a negative adsorption energy for *CO while having a positive adsorption energy for *H and is therefore unique in its ability to catalyze the ECO_2_R reaction [41]. This balance allows Cu to optimize the binding energies of reaction intermediates, enabling the formation of various C_1_ and C_2+_ products, including hydrocarbons and oxygenates such as CH_3_OH [42]. As a result, Cu has been the focus of extensive research over the past three decades, with numerous experimental and theoretical approaches aimed at enhancing its catalytic activity and selectivity, using pure Cu and Cu modified with other elements [1, 43, 44]. Despite these tremendous efforts, the activity and selectivity of most Cu‐based catalysts for producing CH_3_OH remain low, and further research is absolutely necessary both in terms of the catalyst design and the reaction conditions [45]. Here a summary of Cu‐based electrocatalysts, ranging from Cu nanoparticles to oxide‐derived Cu, alloys, intermetallic phases, and metal‐organic framework (MOF)‐based materials, evaluated under varying operating conditions, is presented in Table 1, ensuring a quicker follow‐up for the reader simultaneously with the detailed description of state‐of‐the‐art Cu‐based materials before diving into the elucidation of the reaction mechanism followed by the deactivation mechanism of Cu and factors affecting the CH_3_OH selectivity.

**TABLE 1:** Summary of state‐of‐the‐art Cu‐based catalysts for ECO_2_R to CH_3_OH.

Electrocatalyst	Parameters					
	Reactor	Electrolyte	E_w_ (V) vs RHE	*j* mA·cm^−2^	FE % CH_3_OH	Refs.
Cu_2_O NPs	H‐cell	KHCO_3_	−0.5	−7.8	47.5	[26]
Cu_2_O	Flow cell	0.5 M KHCO_3_	−1.39 (V_Ag/AgCl_)	−10	42.3	[46]
Cu Nanowires	H‐cell	1 M KHCO_3_	−0.5	—	3.1	[47]
Cu Nanosheets	H‐cell	1 M KHCO_3_	−0.5	—	17	[47]
Cu Nanoflowers	H‐cell	1 M KHCO_3_	−0.5	—	15.8	[47]
Cu sandwich	H‐cell	1 M KHCO_3_	−0.5	−1.2	24	[48]
Cu_2_O‐MWCNTs	H‐cell	0.5 M NaHCO_3_	−0.8	−7.5	38	[49]
Cu_2_O thin films	3‐electrode cell	0.5 M KHCO_3_	−1.25 (VS_SCE_)	—	38	[50]
Nano Cu‐Ag/Graphite	H‐cell	0.5 M KHCO_3_	−0.475 (V_Cell_)	∼ −30	64.7	[51]
Cu_2_NCN	H‐cell	0.5 M KHCO_3_	−0.67	−5.1	64	[32]
Cu_2_NCN	MEA cell	0.5 M KHCO_3_	3.4 (E_cell_)	−92.3	70	[32]
CuGa_2_	H‐cell	0.5 M KHCO_3_	−0.3	—	77	[36]
CuGa_2_	Flow‐cell	1M KOH	−0.3	−21.4	78	[36]
Cu_2_O/ZnO	H‐cell	0.5 M KHCO_3_	−1.39 (V_Ag/AgCl_)	−3.17	17.7	[52]
CuBi_12_	Flow‐cell	0.5 M KHCO_3_	—	−10	18.2	[53]
Cu/TiO_2_/NG	Flow‐cell	0.2 M KI	−0.20	−0.061	19.5	[54]
Cu_2_O‐CuO/Ni foam	H‐cell	0.5 M KHCO_3_ +10 mM Py + HCl	−1.3	−33	6.46	[55]
Pd_83_Cu_17_ aerogel	H‐cell	[Bmim] [BF_4_]: H_2_O (1:3)	−2.1 (V_Ag/AgCl_)	−31.8	80	[56, 57]
Cu_1.63_Se (1/3)	H‐cell	30% [Bmim] [PF_6_]/MeCN/ H_2_O	−2.1 (V_Ag/AgCl_)	−41.5	77.6	[35]
CuZn DTA	Flow‐cell	0.5 M KHCO_3_	−0.63	−10	3.4	[35]
Cu_2_O_(OL‐MH)_/ Ppy	3‐electrode cell	0.5 M KHCO_3_	−0.85	—	93	[58]
Cu/Cu_2_P_2_O_7_	H‐cell	0.1 M KHCO_3_	−1.2	4–5	48.8	[12]
Cu/Cu_2_P_2_O_7_	H‐cell	0.1 M CsHCO_3_	−1.1	7–8	70.1	[12]
Cu/Cu_2_P_2_O_7_	Flow‐cell	1 M KHCO_3_	−1.0	113.3	45.6	[12]
Cu/Cu_2_P_2_O_7_	Flow‐cell	1 M CsHCO_3_	−1.0	145.4	65.7	[12]
Cu_0.8_ML/THH Pd NCs	3‐electrode cell	0.5 M NaHCO_3_	−0.46	—	19.5	[59]
Cu_SAs_/TCNFs	3‐electrode cell	0.1 M KHCO_3_	−0.9	−93	44	[34]

### 2.1 Copper‐ and Cu‐Oxide Derived Catalysts

The unique properties of Cu and its ability to adjust the product selectivity in ECO_2_R with oxidation state allow to promote a wide range of reactions to produce numerous gaseous and liquid phase products such as H_2_, CO, CH_4_, HCOO^−^, C_2_H_4_, C_2_H_5_OH, etc., enhancing the activity and selectivity for CH_3_OH production during the ECO_2_R, especially of monometallic Cu, remains a key challenge [27, 60, 61, 62]. Recently the improving effect toward CH_3_OH selectivity was observed while investigating the catalytic performance of a Cu(111) surface and a Cu/G catalyst for the ECO_2_R. Experimental results and Density Functional Theory (DFT) calculations showed that CH_4_ production is relatively easier on the Cu(111) surface, whereas CH_3_OH production is facilitated on the Cu/G catalyst [63]. Subsequently, multi‐walled carbon nanotubes (MWCNTs) were investigated by impregnating with varying loadings of Cu_2_O incorporated at defect sites in the MWCNT matrix. The incorporation of Cu_2_O into the MWCNTs not only improves the electronic properties and increases the active surface area but also increases the accessibility of the reactants to the active sites within the pores. During electrochemical testing, a loading of 30 % Cu_2_O in Cu_2_O‐MWCNTs exhibits the highest current density of − 0.0075 A·cm^−2^ and a Faradaic Efficiency (FE) for CH_3_OH of 38 % at − 0.8 V vs Ag/AgCl with stability over 1200 s [49]. Also, monometallic Cu oxides show high selectivity for CH_3_OH, like metallic Cu, especially when stabilized in specific oxidation states [26, 55].

**FIGURE 3:**
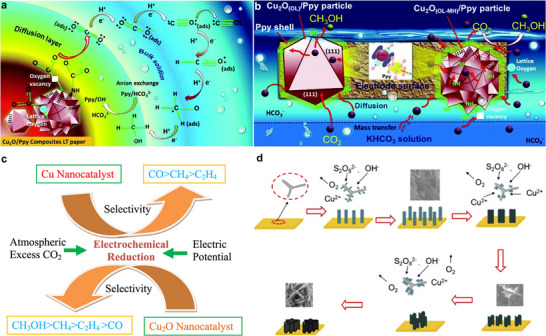
(a) Illustrated mechanism for the formation of CH_3_OH and CO on the Cu_2_O_(OL‐MH)_/Ppy surface. (b) Scheme for the diffusion and mass transport processes at the Cu_2_O_(OL‐MH)_/Ppy particle‐coated LT paper. Modified with permission of Ref. [58]. Copyright 2018, The Royal Society of Chemistry. (c) Schematic representation of the influencing factors during ECO_2_R for selectivity and activity for CH_3_OH production with Cu and Cu_2_O materials. Reproduced with permission of Ref. [26]. Copyright 2019, Elsevier. (d) Illustrative mechanism of the synthesized nanostructured self‐supporting Cu electrode. Reproduced with permission of Ref. [47]. Copyright 2019, Elsevier.

**FIGURE 4:**
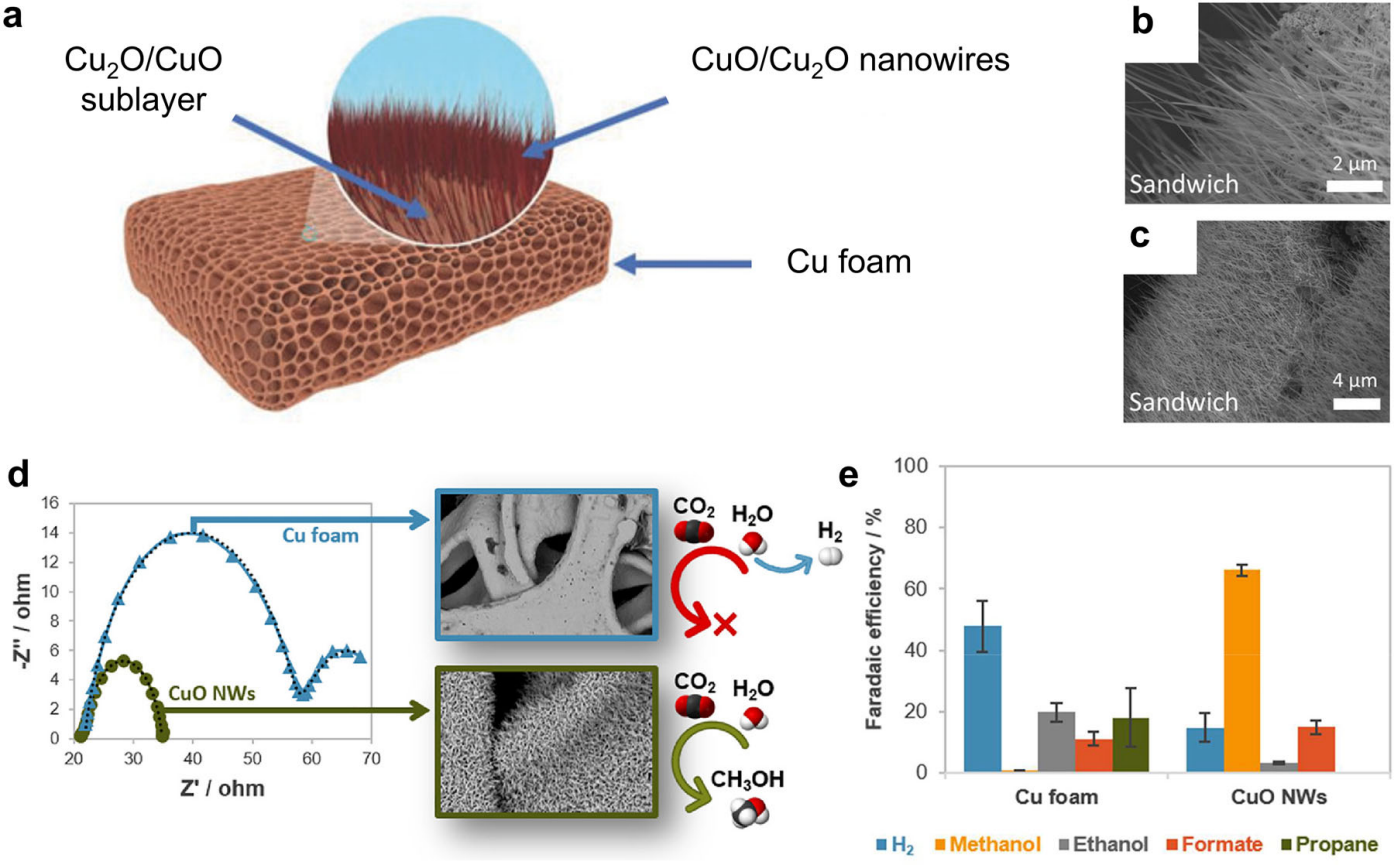
(a) Illustration of the Cu sandwich electrodes, and the corresponding SEM images (b,c) showing the high density of nanowires on the surface. Modified with permission of Ref. [48]. Copyright 2018, John Wiley & Sons, Inc. (d) Schematic comparison of Cu foam and Cu NWs in relation to their charge transfer resistance and their ability to effectively drive the ECO_2_R to CH_3_OH. (e) Faradaic efficiencies of Cu foam and CuO NWs, attained at a continuous current density of ‐45 mA·cm^−2^. Modified with permission of Ref. [28]. Copyright 2022, Elsevier.

In an earlier study, three different types of Cu oxide electrodes were prepared by oxidizing Cu‐foil in an air furnace, by electrochemical oxidation, and electrodeposition on stainless steel substrates, all with mixed oxidation states (Cu_2_O, Cu_4_O_3_, and CuO). The highest CO_2_‐to‐CH_3_OH conversion was observed from thin films by electrodeposition with rates ranging from 10 up to 43 µmol·cm^−2^·h^−1^ and FE up to 38 %, while yields from anodized Cu electrodes ranged from 0.9 to 1.5 µmol·cm^−2^·h^−1^, and yields from the air‐oxidized Cu electrodes were even less and ranged from 0.08 to 0.9 µmol·cm^−2^·h^−1^, indicating that especially Cu_2_O with increased Cu^+^, has a key role in enhancing the activity [50]. A special role of Cu_2_O was also ascribed in the cuprous oxide/polypyrrole (Cu_2_O/Ppy) with an octahedral and icosahedral (microflowers) structure, prepared on linen texture (LT) papers for the selective ECO_2_R to CH_3_OH (Figure 3). The prepared Cu_2_O_(OL‐MH)_/Ppy particles exhibited high activity at a potential of − 0.85 V vs RHE with a ${\mathrm{FE}}_{{\mathrm{CH}}_{3}\mathrm{OH}}$ of 93 ± 1.2 % and a formation rate of r_CH3OH_ = 1.61 ± 0.02 µmol·m^−2^·s^−1^. It was observed that the Cu_2_O particles did not show any sign of corrosion, dissolution, or structural or crystal facet changes. Further, the Cu_2_O particles were protected by the Ppy shell, stabilizing the catalytic system [58]. Synthesizing and stabilizing the morphology, besides the oxidation state of Cu in the respective Cu oxide system, is another critical factor in facilitating efficient CH_3_OH production. Studying the effect of morphology on the catalytic ECO_2_R‐CH_3_OH activity, several nanostructured self‐supporting Cu electrodes have been in situ prepared as nanowires (CuNW), nanosheets (CuNS), and nanoflowers (CuNF). The highest activity for CH_3_OH production was observed using the CuNS electrode with a FE of 12.1 % (total FE_CH3OH_ = 86.9 % with other products such as CH_3_CH_2_OH, CH_3_COO^−^, CH_4_, CH_3_CH_3,_ etc.) at − 0.4 V vs RHE, credited to its nanosheet morphology that was able to better stabilize the intermediate state products [47]. These observations have been backed up by several other studies. When comparing CuO nanowires (NWs) with bare Cu foam, the competing hydrogen evolution reaction (HER) in ECO_2_R to efficiently produce CH_3_OH could be significantly suppressed using CuO NWs. The developed catalyst was able to produce CH_3_OH selectively with an FE of 66.4 % and energy efficiency of 31.2 %, yielding 1.27·10^−4^ mol·m^−2^·s^−1^ of CH_3_OH [28]. A unique 3D heterostructured Cu electrode (Cu sandwich) was synthesized from commercially available Cu foam, with the bare Cu foam. Only the Cu sandwich electrode was able to convert CO_2_ to CH_3_OH with a FE_CH3OH_ of 24 % at − 0.5 V vs RHE and to CH_3_CH_2_OH with a FE_CH3CH2OH_ of 31 % producing a current density of − 1.25 mA·cm^−2^ at a potential of − 0.3 V vs RHE, keeping stable performance for 18 h (Figure 4) [48]. Another very systematic study on pure Cu oxide‐based cathode materials for improved ECO_2_R to alcohols was recently reported. Synthesizing different nanosized Cu_2_O catalysts using a template‐assisted hydrothermal synthesis process, four species, Cu_2_O‐c (cubic structure enclosed with (100) facets), Cu_2_O‐o (octahedron structure enclosed with (111) facets), Cu_2_O‐t (truncated octahedron structure enclosed with both (100) and (111) facets), and Cu_2_O‐u (urchin‐like structure with (211) and (111) facets), were obtained.

Investigating their ECO_2_R to CH_3_OH properties, the following order of alcohol selectivity was observed: Cu_2_O‐t < Cu_2_O‐u < Cu_2_O‐c < Cu_2_O‐o. The highest FE_CH3OH_ measured for Cu_2_O‐o was 4.9 % at a potential of − 0.3 V vs RHE with a high partial current density of − 0.51 mA·cm^−2^, and good long‐term stability [64]. Comparing the ECO_2_R properties of Cu chalcogenides (Cu, CuO, Cu_2_O, CuS, Cu_2_S, CuSe, Cu_2_Se with Cu_2−_ *_x_* Se), an interesting insight was gained related to the cationic effect on the activity of Cu. By tuning the Se ratios in their Cu_2−_ *_x_* Se, ranging from Cu_1.60_Se to Cu_1.64_Se, especially the Cu_1.63_Se(1/3) electrodes showed the best performance at a current density of − 41.5 mA·cm^−2^ with FE_CH3OH_ of 77.6 % at − 2.1 V vs Ag/Ag^+^. Based on their electrochemical analysis, the best cooperative effect for the formation of CH_3_OH with enhanced production was observed when the O or S atom was replaced by the Se atom in the electrocatalysts [35].

**FIGURE 5:**
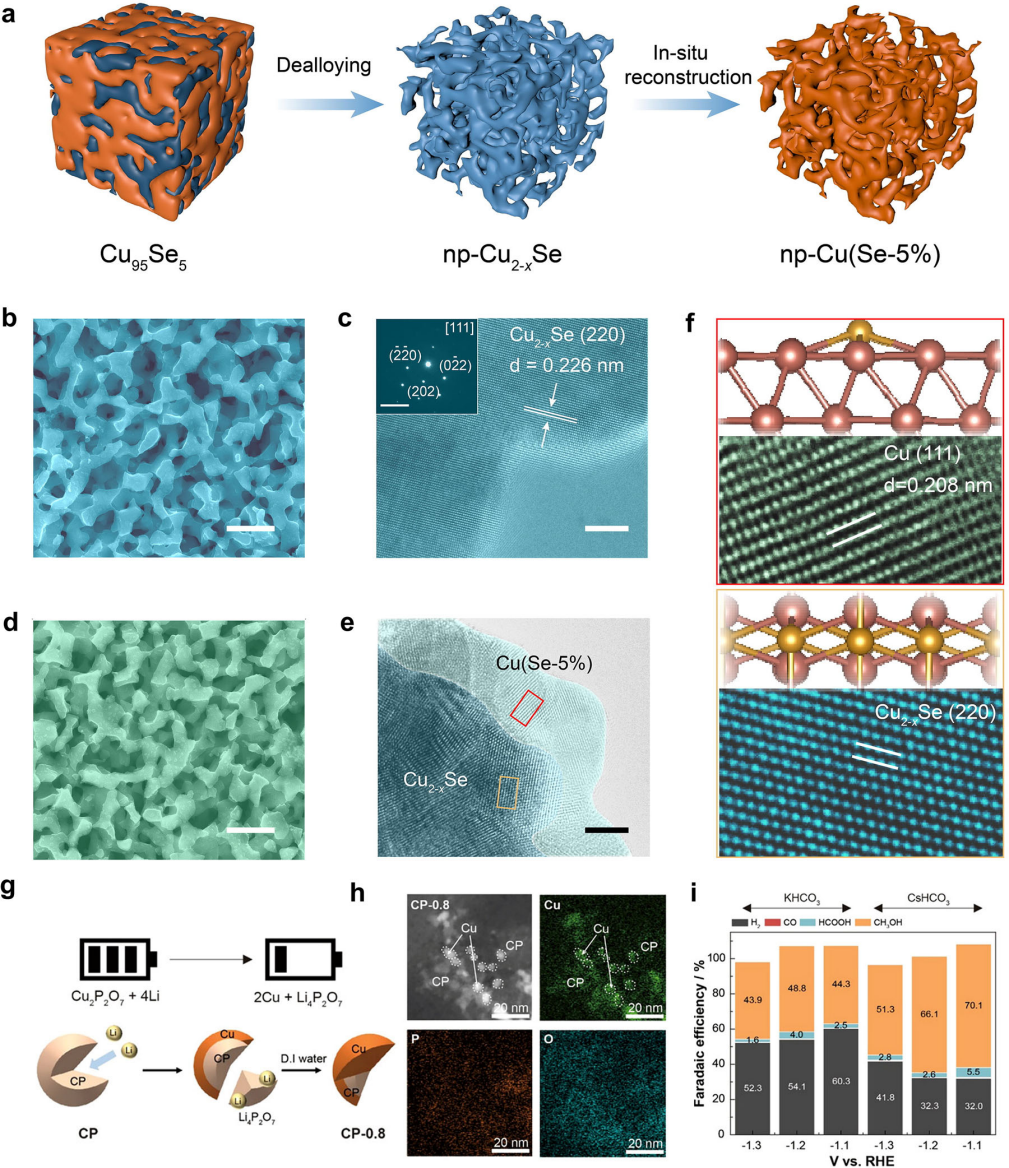
(a) Schematic illustration for the preparation and in situ reconstruction of the np‐Cu(Se‐5%). (b) Scanning electron microscopy SEM image of np‐Cu_2−_ *_x_* Se electrode, with respective transmission electron microscopy (TEM) image (c), and the inset shows the SAED pattern. (d) SEM image of np‐Cu(Se‐5%) electrode with respective TEM image (e) showing Cu(Se‐5%) nanocrystals supported on the surface of np‐Cu_2−_ *_x_* Se phase. Representation of the crystalline structure and HRTEM image of Cu(Se‐5%) nanocrystals (f) and np‐Cu_2−_ *_x_* Se (e). Modified with permission of Ref. [65]. Copyright 2022, Elsevier. (g) Illustration of the synthetic pathway of the Cu/CP hybrid catalysts. (h) Elemental mapping images of CP‐0.8. and (i) the electrochemical performance of CP‐0.8 in 0.1 M KHCO_3_ or 0.1 M CsHCO_3_ for the production of CH_3_OH at −1.2 V versus RHE. Modified with permission of Ref. [31]. Copyright 2025, John Wiley & Sons, Inc.

Nanoporous Cu_2−_ *_x_* Se (np‐Cu_2−_ *_x_* Se) showed similar activity and selectivity and exhibited high selectivity for CH_3_OH with FE_CH3OH_ of 58 % at − 0.5 V vs RHE, much higher than pure np‐Cu (Figure 5) [65]. The influence of cations on Cu associated with the change of oxidation state and selectivity was also observed while investigating a Cu‐ and phosphate‐based hybrid catalyst for selective ECO_2_R to CH_3_OH. A Cu_2_P_2_O_7_ (CP) from a Li‐ion can was electrochemically transformed into a Cu/CuP nanocomposite with precise composition. While the composition of Cu and CP and the pH, as well as the type of the electrolyte, played a vital role for efficient and selective CH_3_OH production, especially the CP‐0.8 exhibited a high FE of 50–70 % and demonstrated very high partial current densities exceeding − 100 mA·cm^−2^ in a gas diffusion electrode (GDE) setup [31].

### 2.2 Cu‐Based Bimetallic and Composite Systems

While Cu alone tends to produce a combination of hydrocarbons and oxygenates in ECO_2_R, researchers have found that combining it with other materials in the form of composites or heterostructures can drive the reaction more toward the formation of CH_3_OH. Cu/TiO_2_ nanoparticles (NPs) decorated onto nitrogen‐doped graphene (Cu/TiO_2_/NG) demonstrated selectivity toward CH_3_OH and CH_3_CH_2_OH, producing CH_3_OH with a (FE) of 19.5 %. The enhanced catalytic activity compared to bare Cu/NG was ascribed to the synergistic effect among Cu, TiO_2,_ and NG, which seems to enhance mass diffusion, and TiO_2_ as a co‐catalyst with its strong redox ability facilitates ECO_2_R, stabilizes intermediates, and reduces the electrolysis potential of the reaction [54]. A similar approach was conducted very recently by mixing graphite, Ag powder, and Cu NPs and pressing a mold of thin cylindrical disk electrodes of various compositions. While mixing Cu with Ag had a negative effect on the overall activity and selectivity, and only pure nano‐Cu electrodes were most efficient for CH_3_OH and CH_3_CH_2_OH production, it was noted that enhanced activity was observed in the Cu‐graphite composites. Especially E_21_Cu_79_C (78.9 wt.% graphite and 21.1 wt.% of nano‐Cu) demonstrated a decreased electrolysis potential of 0.078 V vs Ag/AgCl, and FE_CH3OH_ of 64.71 %, FE_CH3CH2OH_ of 99.25 % [51].

In early works, it was also found that impurities of Fe and Zn found in Cu seem to have an enhancing effect on the ECO_2_R to CH_3_OH [66]. Also, mixed ruthenium‐titanium oxide electrodes modified with small amounts of Cu increased the efficiency of ECO_2_R with FE_CH3OH_ of 29.8 % [67]. Comparable observations have been made for Cu and Cd‐modified RuO_x_ electrodes, which showed a drastic improvement in the CO_2_‐to‐CH_3_OH conversion compared to bare RuO_x_ electrodes [68]. In this regard, Cu and Zn systems have been extensively studied, and several systems have demonstrated that combining both elements is a promising step toward efficient CO_2_‐to‐CH_3_OH conversion. Cu nanoclusters on single‐crystal ZnO electrodes had improved selectivity to produce CH_3_OH when compared to pure Cu electrodes. While no significant differences in the current densities between bare Cu and Cu/ZnO were observed, the change of product distribution toward C_1_ and C_2_ alcohols rather than hydrocarbons or H_2_ was obvious, improving the FE_CH3OH_ from 0.1 % for Cu(111) to 2.8 % for Cu/ZnO [69]. Studying the correlation of selectivity and activity between Zn and Cu for the ECO_2_R to CH_3_OH process, the performance of Cu_2_O and Cu_2_O/ZnO for the continuous ECO_2_R into CH_3_OH was investigated. Different loadings of Cu_2_O particles and Cu_2_O/ZnO weight ratios had a tremendous impact on the rate of CH_3_OH production. The most stable rate of CH_3_OH formation with *r* = 3.17 × 10^−5^ mol·m^−2^·s^−1^ was achieved with Cu_2_O/ZnO (1:1)‐based electrodes with a FE of 17.7 %, stable for 5 h. Furthermore, it was revealed that increasing Cu(I) content while increasing the catalytic loading from 0.5 to 1 mg·cm^−2^ increases the CH_3_OH production and efficiency [52]. During GDE experiments, Cu_2_O/ZnO–GDEs were more stable (over 20 h) than pure Cu_2_O electrodes, exhibiting slight deactivation over time [46]. The same group investigated the ECO_2_R to CH_3_OH at Cu_2_O/ZnO with GDE in different pyridine‐based electrolytes and different concentrations. It was shown that using pyridine‐based co‐catalysts lowered the electrolysis potential for the ECO_2_R to CH_3_OH, reaching formation rate *r* = 4.42 µmol·m^−2^·s^−1^ and FE_CH3OH_ of = 25.6 % [70].

Using Cu‐Zn‐based metal‐organic porous materials, specifically MOF‐based materials such as [Cu_3_(µ_6_‐C_9_H_3_O_6_)_2_]*_n_* (HKUST‐1) and a mesoporous metal‐organic aerogel (MOA) [Cu_0.6_Zn_0.4_(µ‐C_2_H_2_N_2_S_2_)]*_n_* (CuZnDTA) as ECO_2_R catalyst to produce CH_3_OH, the HKUST‐1 and CuZnDTA demonstrated FEs of 15.9 and 9.9 %, at a potential of − 0.9 and − 1.25 V vs RHE, respectively, reaching a current density of − 10 mA·cm^−2^. In comparison, although the catalysts had higher selectivity for CH_3_CH_2_OH than CH_3_OH (FE_CH3OH_ of 5.6 < FE_CH3CH2OH_ of 10.3 for HKUST‐1 and FE_CH3OH_ of 3.4 < FE_CH3CH2OH_ of 6.5 using CuZnDTA), they were found to be more efficient for the production of C_2+_ products [71]. Aligning with these findings, Zn‐doped 2D‐Nanosheet of Cu_2_(OH)_3_(NO_3_) was found to produce CH_3_OH in low potential regions (>− 0.9 V vs RHE), but CH_3_CH_2_OH was primarily the preferred liquid product. Since CH_3_OH was only observed as a side product in trace amounts, it was concluded that Zn in Cu‐based compounds is primarily useful for CH_3_CH_2_OH production [72].

**FIGURE 6:**
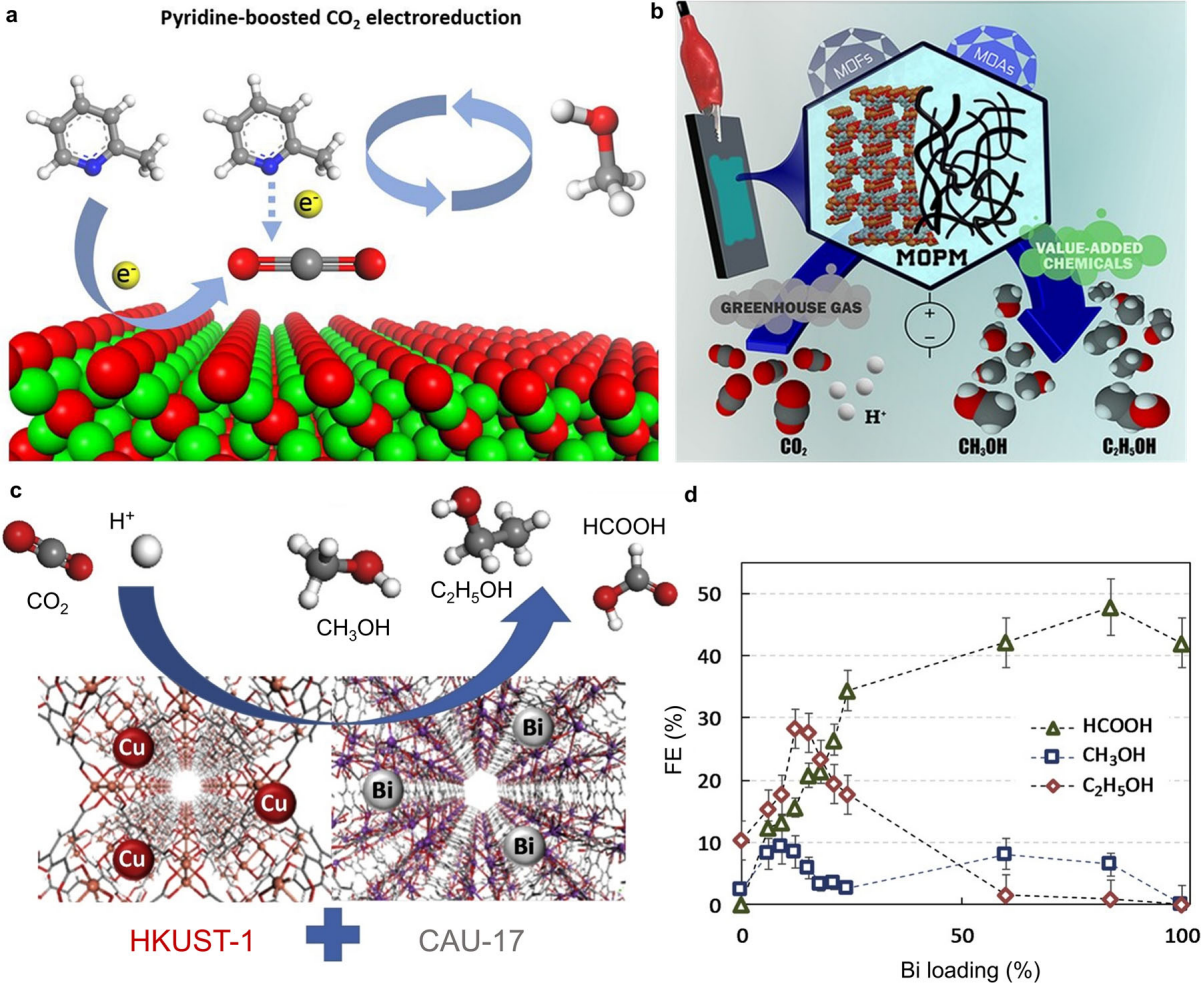
(a) Illustrative effect of pyridine‐based soluble co‐catalysts on Cu_2_O/ZnO‐based surfaces to enhance CH_3_OH selectivity during ECO_2_R. Reproduced with permission of Ref. [70]. Copyright 2017, John Wiley & Sons, Inc. (b) Scheme for metal–organic porous materials (MOPM) as effective electrocatalysts for the ECO_2_R to alcohols. Reproduced with permission of Ref. [71]. Copyright 2016, Elsevier. (c) Scheme of the Cu loaded HKUST‐1 and Bi‐loaded the CAU‐17 acting as efficient ECO_2_R catalysts for value‐added C_1_ and C_2_ products. (d) Graphical representation of the correlation of Bi loading and resulting FE for respective products during ECO_2_R. Modified with permission of Ref. [53]. Copyright 2019, Elsevier.

A couple of years later, the same group published an additional work on Cu‐based ECO_2_R using a Cu(II) and Bi(III)‐based MOF (HKUST‐1 and CAU‐17). Their bimetallic materials supported on porous carbon paper exhibited a favorable continuous ECO_2_R to CH_3_OH and CH_3_CH_2_OH, together with HCOO^−^ and gas‐phase products (i.e., H_2_, CO, and C_2_H_4_). This was the first time that, including bismuth as a co‐catalyst, the electrolysis potential required for ECO_2_R to produce CH_3_OH could be significantly lowered. The highest reaction rates for ECO_2_R to CH_3_OH (r_CH3OH_ = 29.7 µmol·m^−2^·s^−1^ and a ${\mathrm{FE}}_{{\mathrm{CH}}_{3}\mathrm{OH}}$ of 8.6 %, and r_CH3CH2OH_ = 48.8 µmol·m^−2^·s^−1^ (${\mathrm{FE}}_{{\mathrm{CH}}_{3}\mathrm{OH}}$ of 28.3 %), were achieved at a current density of − 20 mA·cm^−2^, outperforming monometallic Cu and Bi‐based electrodes, stable operation over 5 h (Figure 6a,b). The best values for a more selective ECO_2_R toward the alcohols were observed at a Bi content of 12 % and a current density of j = − 20 mA·cm^−2^. Further, it was concluded from the results that the Cu/Bi‐metal ratio and the applied current density had a crucial role in controlling reaction selectivity [53].

Alloyed and bimetallic combinations of Cu and noble metals have recently gained more and more interest for efficient CH_3_OH conversion. Using hydrogen‐storing materials that are modified with suitable materials can significantly improve the ECO_2_R selectivity toward valuable C_1_ products [73]. Several studies showcase that Ru, Pd, Pt, and Au have a positive effect on the activity of Cu toward CH_3_OH production. Although often the reaction mixtures contained different gaseous and liquid products, with CH_3_OH merely a side product, a positive effect toward alcohol selectivity could be observed [74, 75, 76, 77]. A noteworthy increase toward alcohol selectivity was observed using a porous 3D nanostructured network of CuAu. The Cu_63.9_ Au_36.1_/NCF achieved a FE of 15.9 % for CH_3_OH at − 1.1 V vs SCE, reaching a current density of − 0.85 mA·cm^−2^, and performing significantly better than pure Cu [78]. With the help of a pyridine derivative 4‐(3‐phenoxy‐2,2‐bis(phenoxymethyl)propoxy)pyridine (PYD) that was immobilized within a Cu–Pt alloy, a [PYD]@Cu–Pt composite was synthesized for effective ECO_2_R to CH_3_OH conversion at low electrolysis potentials. The composite showed high and almost pure CH_3_OH production at − 0.6 V vs SCE (µ = − 22 mA cm^−2^) with an FE of 37 % stable production for 22 h and recyclable for 12 times. The selectivity only switched when the working potential was shifted to higher values (− 1.2 V vs SCE). Then, CH_3_CH_2_OH was identified as the major product (FE of 24 %) [79]. The same year, the same group published the [PYD]@Cu–Pd composite for the same reaction. CH_3_OH production was realized with a FE of 26 ± 1 % at − 0.04 V vs RHE. Since no conversion to CH_3_OH was detected without PYD (in the case of Cu, Pd NPs, or Cu–Pd alloy), the generation of CH_3_OH was ascribed to the presence of the pyridinium ring [80]. Pd*_x_* Cu*_y_* aerogels with varying compositions were found to be active for the ECO_2_R to CH_3_OH. Especially, the Pd_83_Cu_17_ aerogel electrode was found to be a very efficient and stable electrocatalyst for the ECO_2_R to CH_3_OH process, demonstrating an FE_CH3OH_ of 80.0 % with a current density of − 31.8 mA·cm^−2^ at − 2.1 V vs Ag/Ag^+^ [56, 57]. In another report combining Pd and Cu to optimize the ECO_2_R to CH_3_OH reduction process, tetrahexahedral Pd nanocrystals (THH Pd NCs) with {310} high‐index facets were covered with Cu layers to tune the selectivity and activity toward alcohols. At low Cu coverage, C_1_ products were favored; at higher Cu coverage, C_2_ selectivity was preferred. This way it was possible to tune the selectivity to reach a high selectivity of CH_3_OH (FE_CH3OH_ of19.5 %) at low Cu coverage, and a high selectivity for CH_3_CH_2_OH (FE_CH3CH2OH_ = 20.4 %) at higher Cu coverage, too high coverage with Cu gave low FE for both CH_3_OH and CH_3_CH_2_OH, similar to pure Cu [81].

**FIGURE 7:**
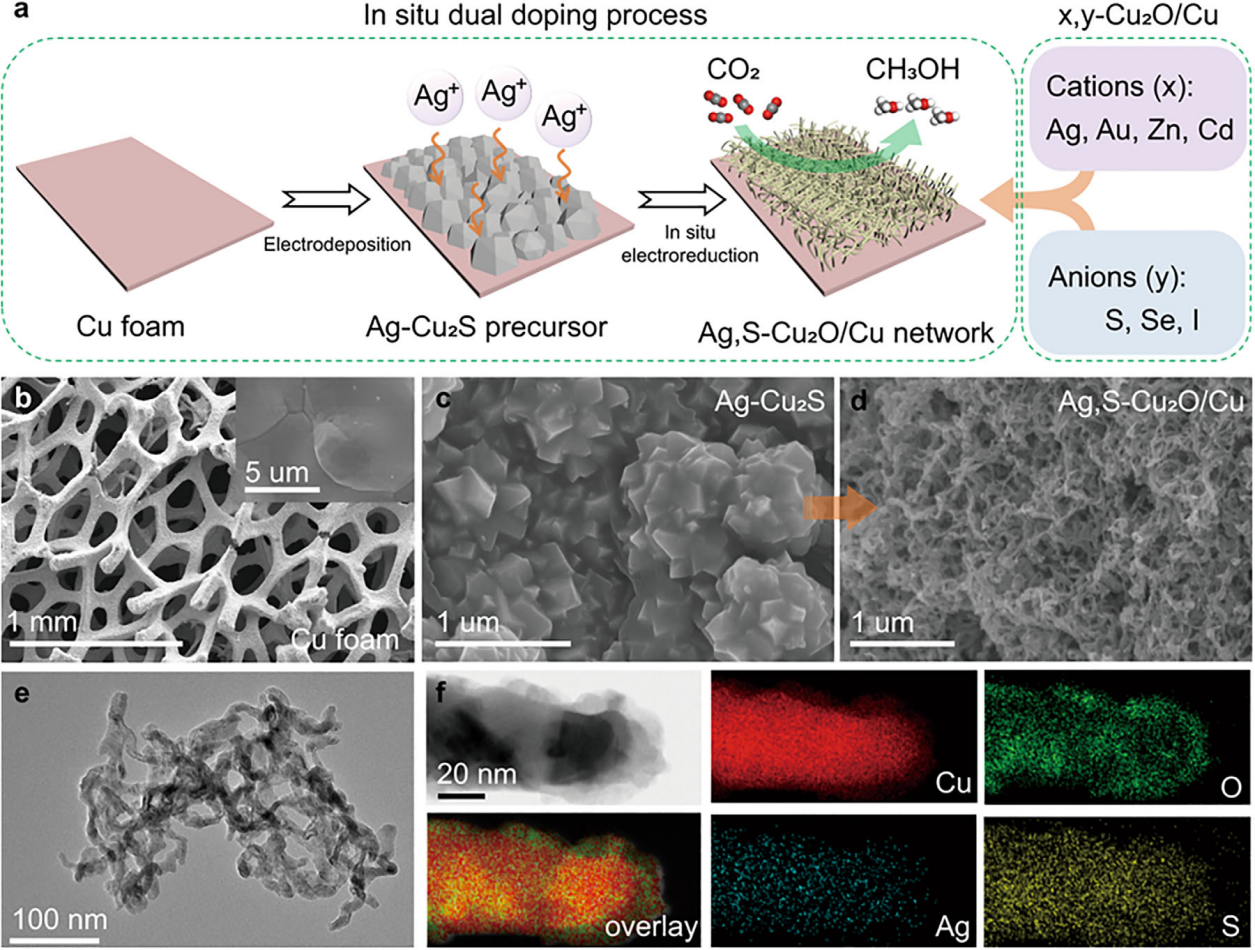
Schematic representation of the synthesis process for the x,y‐Cu_2_O/Cu catalysts. SEM images of (b) bare Cu foam substrate, (c) Ag‐Cu_2_S precursor on Cu foam substrate, and (d) Ag,S‐Cu_2_O/Cu formed at an electroreduction time of 30 min. (e) TEM image of the Ag,S‐Cu_2_O/Cu. (f) Scanning transmission electron microscopy (STEM) and elemental mapping of a typical Ag,S‐Cu_2_O/Cu. Modified with permission of Ref. [37]. Copyright 2022, Springer Nature Limited.

By dual‐doping a Cu_2_O/Cu host structure with various cations (x  =  Ag, Au, Zn, Cd) and anions (y  =  S, Se, I), a series of x,y‐Cu_2_O/Cu structures was synthesized, as illustrated in Figure 7a. The most active species, the Ag, S‐Cu_2_O/Cu, from the in situ transformation of the Ag‐Cu_2_S precursor during electrochemical investigations consisted of a 3D porous morphology with an interconnected network of nanowires, as shown in Figure 7b–f. The good performance of Ag,S‐Cu_2_O/Cu was explained by the change of electronic structure and morphology due to the doping pairs. The S‐anions probably adjusted the adsorption space position of the *CHO intermediate with a lower formation energy barrier, while the Ag‐cations possibly inhibit HER and improve the selectivity, thus enhancing the kinetic CO_2_‐to‐CH_3_OH reduction process [37]. A Cu‐based composite material comprising g‐C_3_N_4_, MoS_2,_ and Cu NPs was prepared and investigated for ECO_2_R to CH_3_OH. The Cu‐g‐C_3_N_4_/MoS_2_ composite demonstrated a higher catalytic efficiency compared to g‐C_3_N_4,_ MoS_2_, Cu‐g‐C_3_N_4,_ and Cu‐MoS_2,_ showing a current density of −195, − 4.9, − 21.4, − 40.1, and − 79.8 mA⋅cm^−2^, respectively. It further showed the highest FE_CH3OH_ of 19.7 % for CH_3_OH and 4.8 % for CH_3_CH_2_OH, enhanced charge transfer, as well as high catalytic stability, without a decline in the current density, for up to 30 h, all beneficial for better performance compared to the reference materials [82].

Two intermetallic compounds formed by Cu and Ga metals (CuGa_2_ and Cu_9_Ga_4_) were investigated for CH_3_OH production at an extremely low applied potential. In neutral conditions, the CuGa_2_ exhibited improved performance for the production of CH_3_OH with an FE_CH3OH_ of 77.26 % at − 0.3 V vs RHE, which was further improved to FE_CH3OH_ of 78 % by using an optimized flow cell under alkaline conditions [36].

### 2.3 Cu‐Based Single‐Atom Catalysts

Lately, single‐atom catalysts (SACs) and single‐alloy atom catalysts (SAACs) have gained enormous attention and become important alternatives in heterogeneous catalysis [83]. By bridging the advantages of homogeneous with heterogeneous catalysts, SACs and SAACs contain high numbers of highly dispersed catalytically active sites on the surface. Therefore, SACs have a nearly 100 % metal atom utilization efficiency and are more advantageous and efficient when compared to nanoparticulate systems [84]. Due to their high activity, selectivity, and stability in catalytic reactions, especially in the field of energy applications, SACs have gained enormous interest and are considered as potential candidates for electrocatalytic applications [85, 86, 87]. Recent results have shown that the SACs were also able to catalyse the reactions of ECO_2_R [88]. Using an Rh‐doped Cu alloy (Rh_1_Cu_4_) to create single atom Rh sites that act as efficient CO to CH_3_OH electrocatalysts, was synthesized to efficiently promote the ECO_2_R. Reaching high current densities of 111.7 ± 12.8 mA·cm^−2^, the Rh_1_Cu_4_ catalyst exhibited an FE_CH3OH_ of 46.2 ± 5.3 %, yielding a r_CH3OH_ = 0.29 µmol·s^−1^·cm^−2^ [89].

**FIGURE 8:**
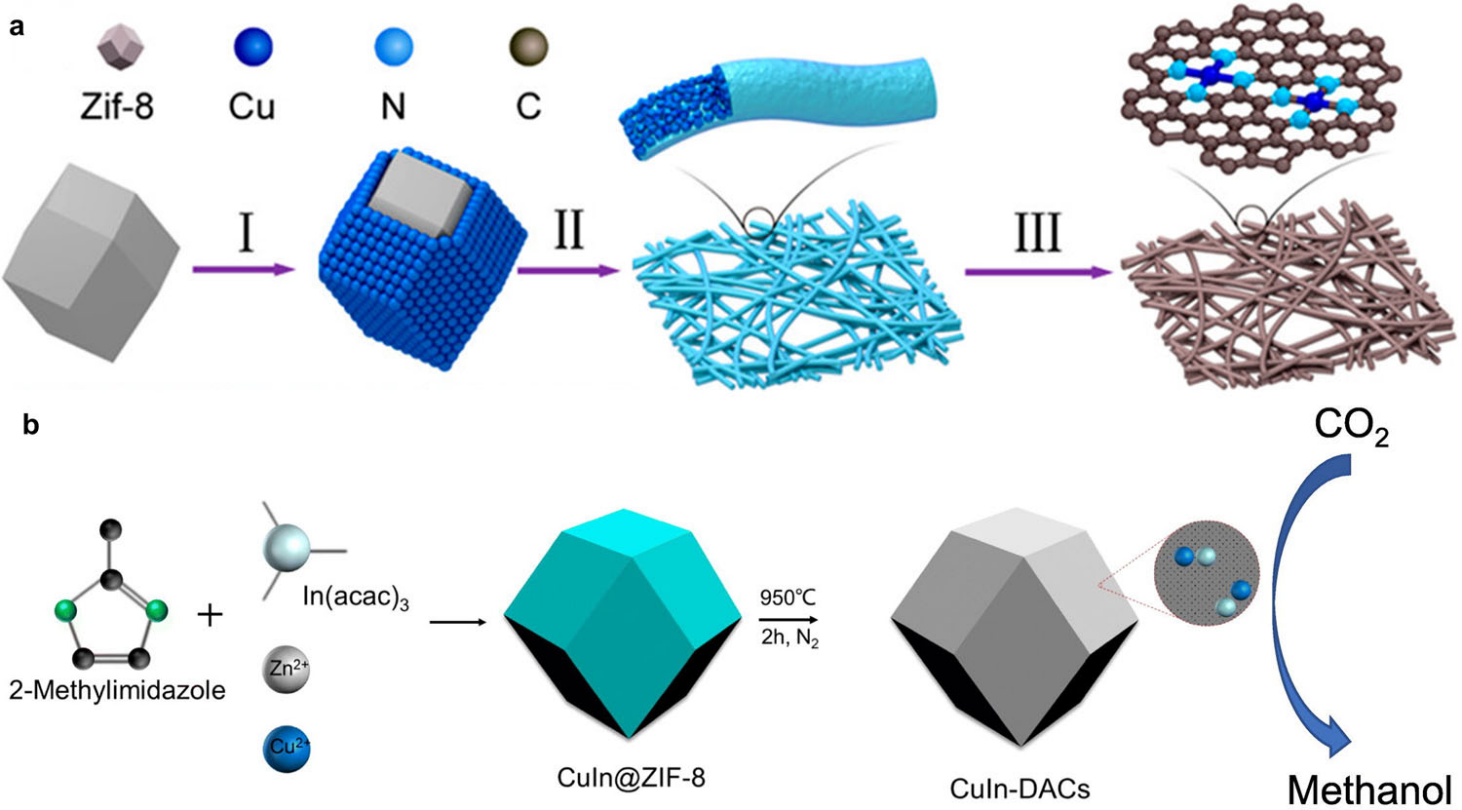
(a) Illustrative Synthesis pathway and digital pictures of the CuSAs/TCNFs membranes. Schematic details of the synthesis procedure of CuSAs/THCF starting with the adsorption of Cu ions (I), followed by the electrospinning of polymer fibers (II), and the carbonization and etching at the end (III). Reprinted with permission of Ref. [34]. Copyright 2019, American Chemical Society. (b) Schematic synthetic approach to attain CuIn‐DACs for selective CH_3_OH in ECO_2_R. Reprinted with permission of Ref. [91]. Copyright 2023, Elsevier.

**FIGURE 9:**
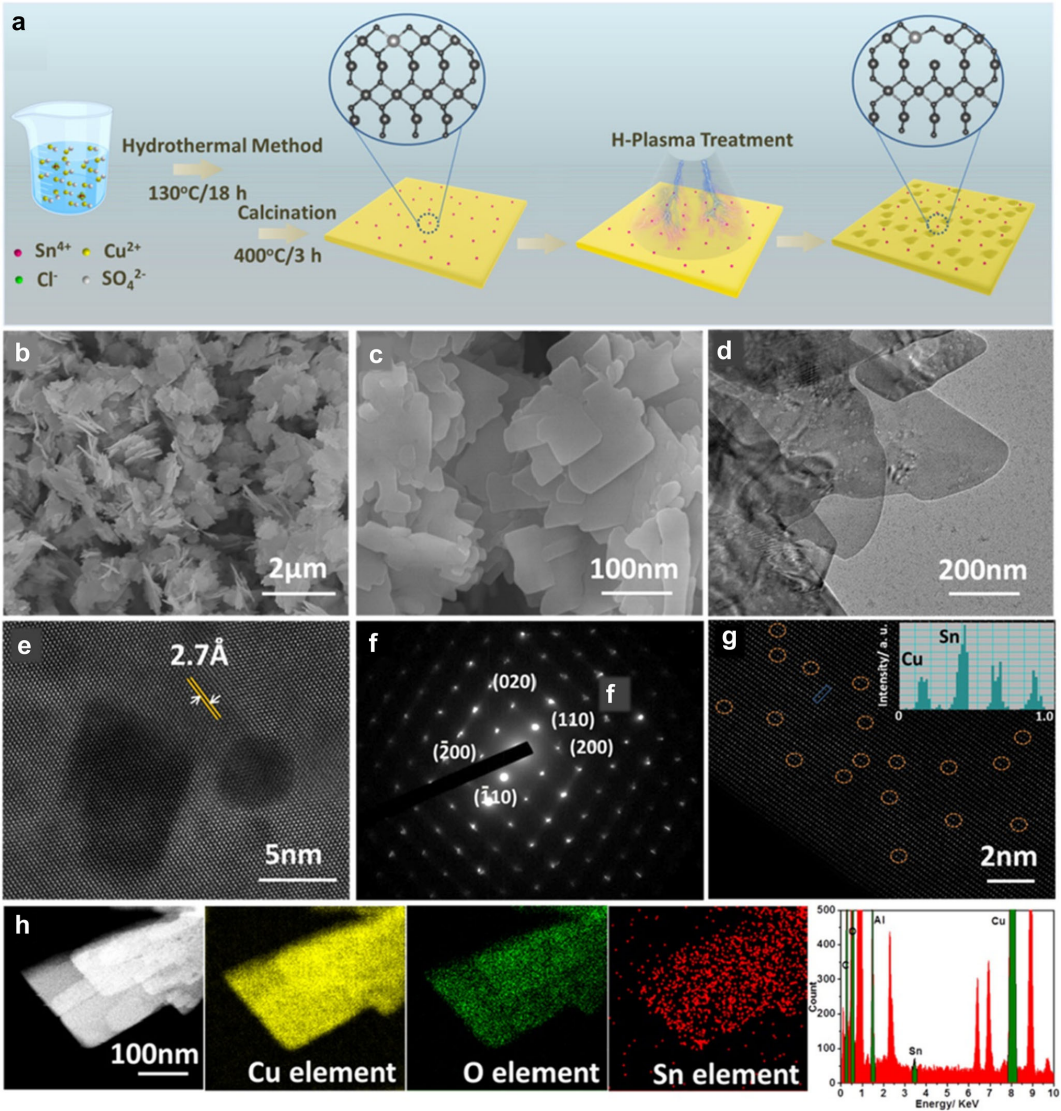
Synthesis and analytics of the Sn_1_/V_o_‐CuO‐90. (a) Schematic for the synthesis of Sn_1_/V_o_‐CuO‐90. (b,c) SEM images of Sn_1_/V_o_‐CuO‐90 at different magnifications. (d) HR‐TEM image and (e) HAADF‐STEM image of Sn_1_/V_o_‐CuO‐90 with (f) related SAED pattern (scale bar:101 nm^−1^). (g) HAADF‐STEM images of Sn_1_/V_o_‐CuO‐90 with atomically dispersed Sn highlighted in circles. (h) EDS mappings of Sn_1_/V_o_‐CuO‐90 and elemental distribution diagram. Modified with permission of Ref. [38]. Copyright 2021, John Wiley & Sons, Inc.

Recently, a facile strategy for the large‐scale synthesis of single‐atom Cu decorated on through‐hole carbon nanofibers (CuSAs/TCNFs) was reported (Figure 8). With its excellent mechanical properties, the CuSAs/TCNFs were directly used as a cathode for the ECO_2_R. CH_3_OH was produced at a rate of 68.4 µmol·m^−2^·s^−1^, with FE_CH3OH_ of 44 % at a current density of − 93 mA·cm^−2^ (@− 0.9 V vs RHE), and the electrode was long‐term stable for 50 h [34]. In the meantime, a SAC‐based on Sn was investigated to utilize its ability to promote ECO_2_R and suppress the HER due to the high electrolysis potentials for the latter process. The Sn_1_/V_o_‐CuO‐90 (Figure 9) exhibited the highest activity with a FE_CH3OH_ of 88.6 % at a current density of 67.0 mA·cm^−2^, remaining stable for 36 h at an applied potential of − 2.0 V vs Ag/Ag^+^ [38]. Single‐atom Cu sites immobilized on MXene layers (SA‐Cu‐MXene), synthesized by etching the quaternary MAX phases (Ti_3_(Al_1–_ *_x_* Cu*_x_*)C_2_), have been successfully described as efficient catalysts for CO to CH_3_OH conversion. After the etching process to remove the Al layers, single‐atom Cu was well‐preserved and immobilized onto MXene (Ti_3_C_2_Cl*_x_*). The SACuMXene exhibits a high FE_CH3OH_ of 59.1 % at − 1.4 V vs RHE, reaching a current density of 21.3 mA·cm^−2^, and even after 30 h of testing, the high current density could be maintained well, with a high FE_CH3OH_ of >58 % [33]. Similarly, a Cu(II) bis‐triazine bipyridine complex supported on carbon black was applied as a catalyst in a polymeric electrolytic reactor‐fuel cell (PER‐FC) type for efficient ECO_2_R to CH_3_OH. Based on their results, the catalyst's selectivity could be influenced by variations in mixing the Cu complex in the proportions 1 %, 2.5 %, 5 %, 10 %, and 20 % in carbon (mass/mass) but still a mixture of products (CH_3_OH, HCOO^−^, HCHO, CO, and CH_4_) was obtained. Only 2.5 % and 5 % Cu complexes on carbon black exhibited optimal loading for the PER‐FC applications, achieving CH_3_OH production from CO_2_ with a FE_CH3OH_ of ∼ 22 % for both compositions. Notably, the 2.5 % Cu complex demonstrated a 26 % higher reaction rate, highlighting its remarkable performance [90]. More recently, a cuprous cyanamide (Cu_2_NCN) crystal featuring single‐atom Cu sites and enhanced electronic delocalization around the Cu centers was developed, which acts as an efficient electrocatalyst for the ECO_2_R to CH_3_OH. Inspired by hard–soft acid‐base–base theory, it was proposed that the ECO_2_R pathways toward CH_3_OH or CH_4_ could be switched by tuning the electron delocalization state of Cu catalytic sites. By designing the Cu_2_NCN, it was possible to enable a system with isolated Cu(I) ions strongly conjugated with NCN^2−^, exhibiting highly delocalized electrons. This way, the Cu_2_NCN catalyst reached a FE_CH3OH_ of 64 %, which was further improved to a FE_CH3OH_ of 70 % when a MEA setup was used [32].

Using a similar approach to introduce a dual atomic catalyst with Cu and Indium (In) into the ZIF‐8 structure, Yuan et al. wanted to improve the performance of SAC‐based Cu catalysts. The attained dual atom catalyst (DAC) CuIn‐DACs successfully inhibited the HER to a minimum during ECO_2_R and produced highly selective CH_3_OH with a FE_CH3OH_ of 55.63 % at an applied potential of − 0.62 V vs RHE, which is much higher than that of the compared SACs [91].

## 3 Mechanistic Insights into Methanol Selectivity

The ECO_2_R to CH_3_OH represents a multi‐step process involving several proton‐coupled electron transfer (PCET) events. This transformation is strongly governed by the catalysts’ surface structure, coordination environment, and local electrochemical conditions [1, 92]. Considering the inherent thermodynamic stability of CO_2_, owing to its strong C═O bonds and low Gibbs free energy, its conversion to CH_3_OH, an energy‐dense product with comparatively higher Gibbs free energy, requires significant energy input. This energy can be supplied through various external sources such as electrical energy, thermal input, or light. The process is further complicated by the high activation barriers associated with the formation of key intermediates, such as the CO_2_•^−^ radical anion, and the large energy gap between the molecular orbitals of CO_2_, thereby necessitating the use of highly selective and efficient electrocatalysts to lower these barriers.

### 3.1 General Reaction Pathways and Selectivity Challenges for CH_3_OH Formation

**FIGURE 10:**
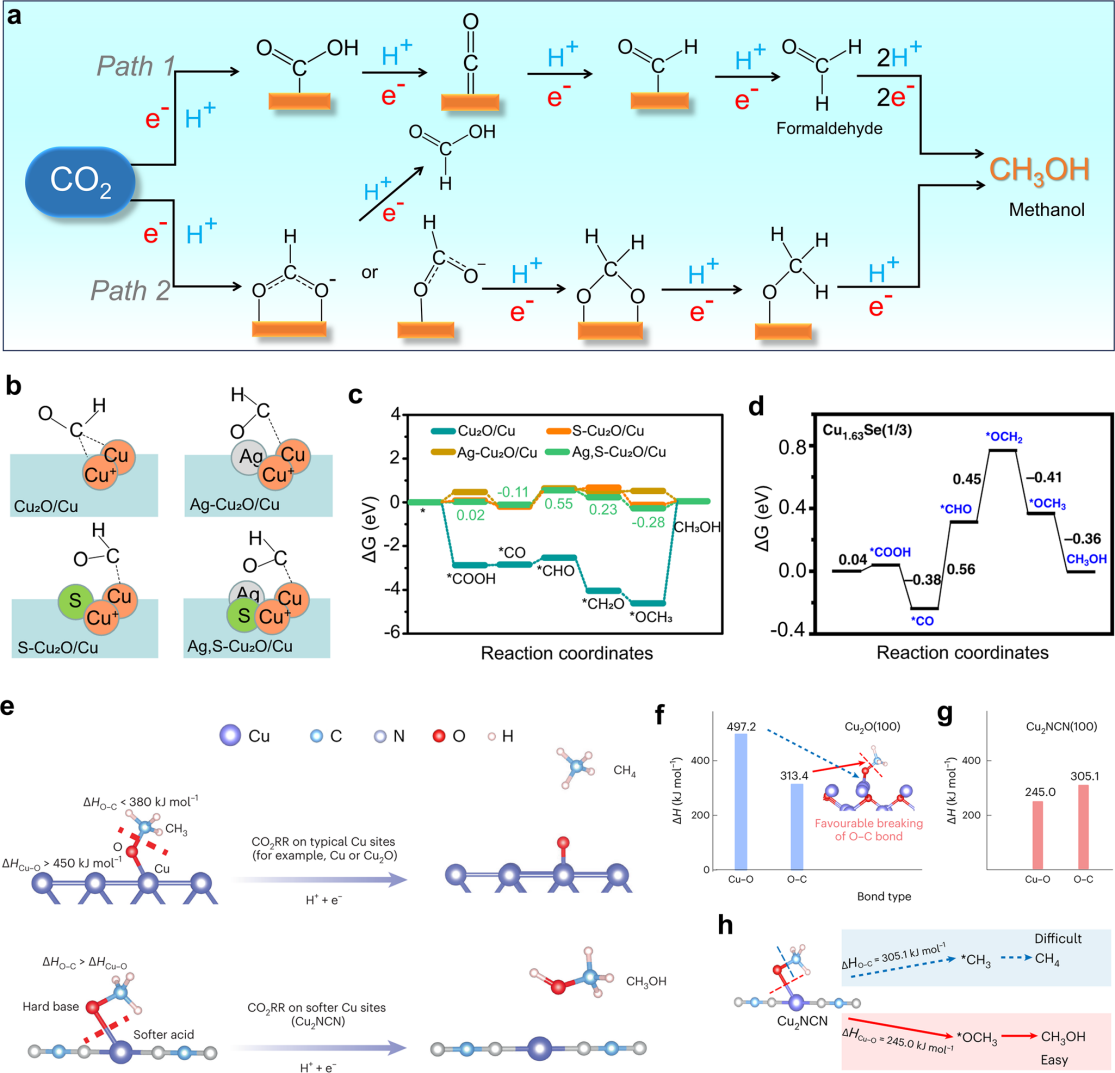
(a) Proposed reaction mechanism for ECO_2_R to CH_3_OH over Cu‐based catalysts, highlighting key proton‐electron transfer steps and intermediate species [36]. Reproduced with permission of Ref. [36]. Copyright 2018, Springer Nature Limited. (b) Computational analysis of ECO_2_R on Ag,S‐doped Cu_2_O/Cu surfaces. Schematic of *CHO intermediate adsorption on various Cu‐based catalysts. (c) Reaction free energy profiles for the proposed CO_2_ to CH_3_OH conversion pathway [37]. Reproduced with permission of Ref. [37]. Copyright 2022, Elsevier B.V. (d) Free energy profile for ECO_2_R on the Cu_1.63_Se(1/3) electrocatalyst for CH_3_OH production [35]. Modified with permission of Ref. [35]. Copyright 2022, Elsevier B.V. (e) Conventional Cu‐based active sites, such as metallic Cu or Cu_2_O, typically exhibit a high bond dissociation enthalpy for the Cu─O bond (ΔH_Cu–O_ > 450 kJ mol^−1^), which is notably stronger than the O─C bond in methoxy species (ΔH_O–C_ < 380 kJ mol^−1^ in OCH_3_). In contrast, catalysts featuring softer acid sites, such as Cu_2_NCN, can lower the Cu─O bond dissociation energy below that of the O─C bond, thereby promoting the selective reduction of CO_2_ toward CH_3_OH instead of CH_4_. Comparison of Cu─O and O─C bond dissociation energies in Cu─*OCH_3_ intermediates on (f) Cu_2_O(100) and (g) Cu_2_NCN(100). (h) Schematic showing the bond cleavage sites directing the reaction pathways toward either CH_4_ or CH_3_OH on Cu_2_NCN [32]. Modified with permission of Ref. [32]. Copyright 2022, Springer Nature Limited.

The ECO_2_R to CH_3_OH proceeds via a complex six‐electron, six‐proton transfer pathway under ambient conditions, typically involving two primary reaction routes: one via CO (*path 1*) as the key intermediate and the other through HCOO^−^ (*path 2*) (Figure 10a) [93, 94]. The ECO_2_R to CH_3_OH formation process suffers from inherently sluggish kinetics and significant competition from HER, which often dominates under aqueous conditions. Despite its thermodynamic favorability, the reduction potential of CO_2_ is ∼ 20 mV, more positive than that of the HER [95]. Moreover, the generated CO intermediate can also participate in competing multi‐carbon product (C_2+_) formation routes, thereby diverting the reaction pathways and decreasing the selectivity of CH_3_OH [96]. In this scenario, the appropriate design of electrocatalysts is extremely crucial to promote selective CH_3_OH formation by stabilizing the desired intermediates and simultaneously suppressing C─C coupling and HER side reactions [97]. Cu‐based catalysts provide a unique balance between binding strength and intermediate stabilization, making them promising candidates for ECO_2_R to CH_3_OH [14, 98]. On Cu, the pathway to CH_3_OH proceeds primarily through the formation of *CO as a key intermediate (following *path 1*), with the selectivity being dictated by the ability of catalyst materials to retain *CO without promoting its desorption or diverting it toward C─C coupling routes that lead to C_2+_ products [99]. Weak *CO binding results in premature desorption as CO is a major product, while excessively strong binding can block active metal sites and hinder subsequent reduction steps. Optimal *CO stabilization allows its protonation to *CHO or *OCH_3_ intermediates, crucial for CH_3_OH formation.

It is important to mention that the formation of *COH intermediates diverts the reaction toward CH_4_, whereas *CHO and *OCH_3_ are more favorable for CH_3_OH production [100, 101]. However, the exact mechanism beyond *CHO remains under debate, with studies suggesting divergent outcomes toward either CH_3_OH or CH_4_ depending on the binding configuration and catalyst properties. In particular, *OCH_3_, identified as an O‐bound intermediate with a surface‐repelling ─CH_3_ group, is a key intermediate species for CH_3_OH formation, where lower OH‐binding energy promotes its reduction to CH_3_OH [14, 102]. Catalyst surfaces with lower O‐affinity are found to inhibit complete C─O bond cleavage, thus favoring alcohol production over hydrocarbon pathways. In contrast, high O‐binding energies promote further reductions toward CH_4_ or C_2+_ products [102]. Overall, achieving CH_3_OH selectivity on Cu requires precise modulation of intermediate binding energies and suppression of parallel HER and C─C coupling pathways through structural and electronic tuning of the catalyst surface.

To provide deeper insights and considering the practical aspects, it is important to relate the mechanistic understanding of methanol formation to strategies for rational design of catalyst materials. As mentioned before, the critical intermediates, especially *CO, *CHO, and *OCH_3,_ act as “selectivity‐determining species” defining whether the reaction produces CH_3_OH or proceeds further toward CH_4_, CO, or C_2_^+^ products [103]. Therefore, tuning the electronic structure and surface coordination environment of the active Cu site can directly influence these intermediate binding energies [35]. For instance, introducing electron‐donating dopants (such as In, Zn, or Ga) or constructing Cu‐oxide interfaces has been shown to optimize *CO stabilization and facilitate its subsequent hydrogenation to *CHO and *OCH_3_, thereby enhancing CH_3_OH selectivity [36, 69, 104]. Similarly, defect or strain engineering can create undercoordinated Cu sites that stabilize *OCH_3_ while suppressing C─C coupling and HER. Beyond catalyst composition, controlling the reaction microenvironment through electrolyte engineering, pH optimization, or tuning the electric double layer can promote PCET pathways that favor methanol over competing products. Integrating insights from DFT‐calculated free energy diagrams and operando spectroscopic evidence enables the identification of rate‐ and selectivity‐determining steps, providing a mechanistic foundation for designing better Cu‐based catalysts with enhanced activity and selectivity toward methanol.

### 3.2 Computational Perspectives for Methanol Formation

Computational studies, including DFT calculations, ab initio molecular dynamics (AIMD), and machine learning (ML) have been widely employed to understand the elementary steps and reaction energetics of ECO_2_R to CH_3_OH. They provide valuable mechanistic insights, identifying key intermediates correlating theoretical predictions with experimental observations to guide the rational design of more selective catalysts [100, 105].

#### 3.2.1 Active Site Determination and Configurations of Adsorbed Intermediate

Ab initio calculations and DFT have systematically investigated the stabilization of key intermediates *CO, *CHO, *CH_2_O, and *CH_3_O on Cu‐based surfaces, including facets (Cu(111), Cu(100), Cu(110)), nanoclusters, and engineered single‐atom systems [106, 107, 108]. These studies predict that electron‐rich Cu sites and specific atomic arrangements, such as twin boundaries and step edges, preferentially anchor *CHO and *CH_2_O, thus facilitating methanol formation over methane or C_2+_ evolution. Notably, solvent‐inclusive DFT calculations demonstrate that solvation minimizes the activation barriers for *CO hydrogenation on certain Cu surfaces, shifting selectivity toward methanol [109, 110]. Previously, DFT calculations have revealed that dual doping with Ag and S in Cu_2_O/Cu catalysts (Figure 10b) modulates the electronic environment of Cu sites, resulting in optimal COOH adsorption energy (ΔG_COOH_ ≈ 0.02 eV) and reduces hydrogenation barrier for *CO to *CHO (0.66 eV), as presented in Figure 10c [37]. This modification facilitates CO_2_ reduction to CH_3_OH while suppressing HER activity. A lowered d‐band center and favorable Bader charge redistribution further confirm enhanced intermediate stabilization and product selectivity, establishing Ag,S‐Cu_2_O/Cu as highly selective CH_3_OH catalysts. Similarly, DFT analysis of Cu_1.63_Se(1/3) demonstrated a thermodynamically favorable *COOH formation pathway via dual Cu─C and Cu─O coordination environment, with a lower free energy than that of Cu_2_Se and CuSe (Figure 10d) [35]. The *CO to *CHO step was found to be the rate‐limiting step (RDS), showing the lowest energy barrier (0.56 eV) on Cu_1.63_Se(1/3), supported by strong *CHO adsorption (Cu─C bond = 1.926 Å), promoting efficient hydrogenation to CH_3_OH [111]. The intrinsic structural distortion present in Cu_1.63_Se(1/3) is a key factor contributing to its superior CH_3_OH selectivity. Recent DFT investigations on Cu_2_NCN reveal that the unique zigzag crystal structure, composed of alternating cyanamide subunits (α and β), introduces distinct electronic features responsible for the CH_3_OH selectivity (Figure 10e) [32]. Compared to Cu_2_O, Cu_2_NCN exhibits reduced partial density of states (PDOS) peak intensities and a significantly lower effective electron mass (0.25 Hartree), indicating enhanced electron delocalization. This delocalization, driven by strong NCN^2−^ coordination, induces cation polarization and produces softer Cu sites. According to the hard–soft acid–base (HSAB) theory, these softer Cu sites bind less strongly to oxygenated species like *OCH_3_, leading to a lower Cu─O bond dissociation enthalpy (245.0 kJ mol^−1^ on Cu_2_NCN vs 497.2 kJ mol^−1^ on Cu_2_O) (Figure 10f,g) [112]. As a result, the catalyst favors the release of *OCH_3_ and promotes CH_3_OH production (Figure 10h). Similarly, Cu_2_NCN(100) also shows a thermodynamic preference for CH_3_OH over CH_4_ due to stabilized *CHO intermediates and lower kinetic barriers, facilitated by its electron‐delocalized structure and weakened Cu─O interactions. Overall, these DFT studies consistently demonstrate that CH_3_OH selectivity on Cu‐based catalysts is strongly governed by the electronic structure of the catalyst surface, nature of intermediate stabilization and Cu─O interaction strength. Tuning the catalyst composition and structure, such as inducing electron delocalization or moderate *CO binding energy can facilitate the reaction pathway toward *CHO formation and promotes subsequent hydrogenation steps, ultimately favoring CH_3_OH formation over CH_4_ or C─C coupled products.

#### 3.2.2 Energetics and Scaling Relationships for Methanol Selectivity

Over the years, computational models have utilized reaction energy profiles and scaling relationships to compare the energetics for the formation of methanol, CO, CH_4_, and C_2+_ production on Cu alloys and hybrid systems [108]. As mentioned earlier, selectivity trends correlate strongly with the binding energy of the crucial intermediates, especially *CO and *CHO, which define the preference for stepwise versus direct hydrogenation routes leading to either methanol or competing products. Data‐driven DFT studies predict that optimal methanol selectivity is obtained when *CO binding is moderate, providing facile hydrogenation to *CHO but minimizing C‐C coupling pathways responsible for C_2+_ formation [102, 113]. DFT‐guided alloying and doping strategies have been shown to have numerous modifications likely to enhance methanol selectivity. Examples include alloying Cu with Ga, Zn, In, or Ni, which tunes *CO and *CHO stabilization via electronic and geometric effects, and minimizing side reactions. Oxide‐derived or phosphate‐modified Cu surfaces provide distinct charge redistribution, boosting *CH_2_O formation [31]. Cu‐based single atom catalysts embedded on nitrogen‐doped carbon or other conducting support further break scaling relations, facilitating methanol formation at lower overpotentials [114].

**FIGURE 11:**
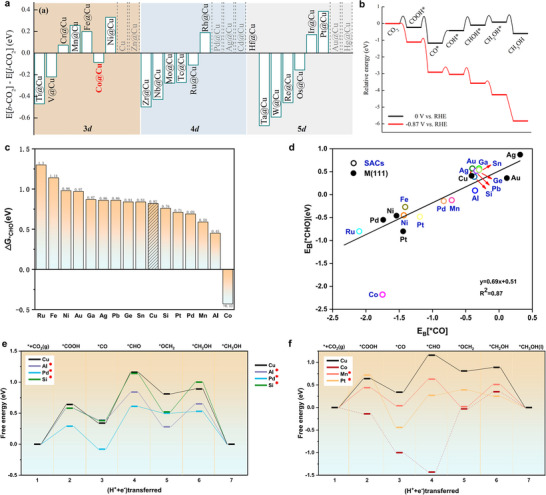
(a) Energy differences between b‐CO_2_ and l‐CO_2_ adsorption configurations on the examined single‐atom alloy (SAA) surfaces. The dashed bars represent the SAA systems where b‐CO_2_ spontaneously converts to l‐CO_2_ during geometric optimization (b) Relative energy profiles for CH_3_OH formation on Co@Cu surfaces at applied potentials of 0 V and − 0.87 V versus RHE. Reproduced with permission of Ref. [115]. Copyright 2019, American Chemical Society. The CH_3_OH formation on various M@Cu catalysts. (c) Energy barriers for *CO to *CHO conversion on M@Cu(111) surfaces. (d) Correlation between *CHO and *CO adsorption energies on M(111) and M@Cu catalysts. (e,f) Energy profiles for CH_3_OH production on M@Cu(111): (e) Al, Pd, Si and (f) Co, Mn, Pt@Cu. Catalysts exhibiting a lower potential‐determining step (PDS) barrier than Cu are indicated with an asterisk. Reproduced with permission from Ref. [106]. Copyright 2023, Cell Press.

Figure 11a screens 26 metal‐doped Cu (111) single‐atom alloys and identifies Co@Cu as one of the most promising systems, where the single Co atom stabilizes bent CO_2_ (b‐CO_2_) and enhances CO_2_ activation. This unique Co‐Cu combination, as shown in Figure 11b, minimizes the electrolysis potential for CH_3_OH formation to − 0.87 V versus RHE, leading to high methanol selectivity over methane [115]. The reduction of *CO to *CHO, a key step toward CH_3_OH formation, is energetically demanding. Figure 11c shows that M@Cu(111) catalysts, particularly Al, Si, Pt, Pd, Mn, and Co, lower this barrier compared to Cu, enhancing activity. Figure 11e,f indicates that catalysts in the adjacent hollow (AD) mode, especially Si@Cu and Al@Cu, favor CH_3_OH formation by reducing the potential‐determining step. Figure 11d reveals that this arises from tuning adsorption energies: weakened *CO binding and strengthened *CHO adsorption lower the *CO → *CHO barrier, exhibiting that composition and adsorption mode in M@Cu SAAs can be optimized for high methanol selectivity [106]. DFT and AIMD studies also predict that coordination environments and lattice strain directly affect the reaction pathway preference.

Computationally guided design has advanced the field beyond empirical trial‐and‐error, integrating microkinetic simulations, atomic‐scale predictions, and data‐driven ML to accelerate the development of next‐generation Cu‐based catalysts. These approaches bridge the gap from theoretical modelling to scalable, industrial ECO_2_R, supporting the realization of carbon‐neutral methanol production at practical rates and selectivity.

## 4 Probing Reaction Mechanisms and Active Sites by In Situ Techniques

Understanding the active sites and reaction mechanisms in ECO_2_R to CH_3_OH on Cu‐based catalysts requires advanced spectroscopic tools. In situ and operando techniques such as X‐ray absorption spectroscopy (XAS), surface‐enhanced Raman spectroscopy (SERS), and Fourier transform infrared spectroscopy (FTIR), and surface‐enhanced infrared absorption spectroscopy (SEIRAS) provide crucial insights into the structural evolution of the catalyst and the identification of key reaction intermediates under working conditions [116, 117, 118, 119, 120].

### 4.1 Surface‐Enhanced Raman Spectroscopy (SERS)

**FIGURE 12:**
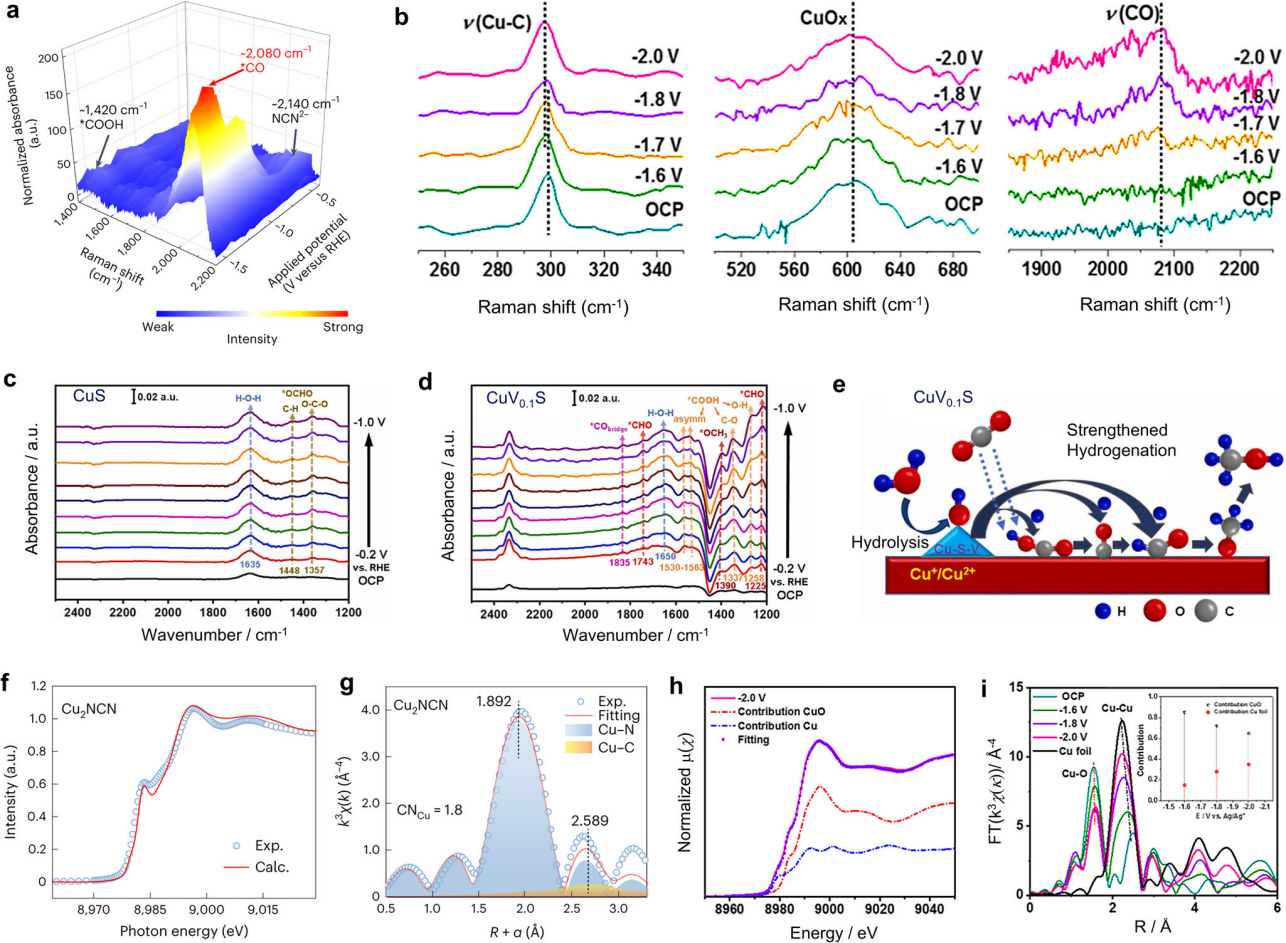
(a) In situ Raman spectroscopy during ECO_2_R on Cu_2_NCN catalyst, recorded across a potential window from − 0.5 to − 1.5 V (vs RHE) [32]. Modified with permission of Ref. [32]. Copyright 2019, American Chemical Society. (b) Potential‐dependent in situ Raman spectra Sn_1_/Vo‐CuO‐90 during ECO_2_R. [38] Potential‐dependent in situ FTIR spectra of (c) CuS and (d) CuV_0.1_S catalysts in CO_2_‐saturated 0.5 M KHCO_3_ electrolyte. Reproduced with permission of Ref. [38]. Copyright 2020, Royal Society of Chemistry. (e) Schematic representation of the ECO_2_R for the CH_3_OH formation pathway over CuV_0.1_S catalyst [124]. Reproduced with permission of Ref. [124]. Copyright 2020, Springer Nature Limited. (f) Comparison of experimental and simulated XANES spectra for Cu_2_NCN, highlighting the agreement between measured data and theoretical calculations. (g) Fourier‐transformed EXAFS spectrum of Cu_2_NCN with fitted curves corresponding to the first coordination shell. Blue and yellow shaded regions indicate the Cu─N and Cu─C scattering paths, respectively [32]. Modified with permission of Ref. [32]. Copyright 2019, American Chemical Society. (h) Linear combination fitting of Cu K‐edge XANES spectra of Sn_1_/V_o_‐CuO‐90 catalyst using reference compounds at an applied potential of − 2.0 V vs Ag/Ag⁺. (i) Fourier‐transformed EXAFS spectra of the Cu K‐edge recorded under varying applied potentials during ECO_2_R on Sn_1_/V_o_‐CuO‐90 catalyst [38]. Reproduced with permission of Ref. [38]. Copyright 2020, Royal Society of Chemistry.

Raman spectroscopy, particularly in SERS, serves as a valuable tool for identifying vibrational signatures of reaction intermediates and monitoring surface dynamics of catalyst materials during ECO_2_R under operando conditions [121, 122, 123]. For the ECO_2_R to CH_3_OH on Cu‐based materials, only a limited number of studies have provided detailed molecular‐level insights into the reaction mechanism and active site dynamics. In situ Raman spectroscopy of CH_3_OH selective Cu_2_NCN catalyst reveals characteristic bands at ∼1420 and ∼735 cm^−1^, corresponding to *COOH and *COO^−^ species, respectively (Figure 12a) [32].

These bands are the characteristics of the intermediate related to CO_2_ activation [125, 126]. A prominent peak at ∼2,080 cm^−^^1^ signifies the formation of adsorbed *CO, a key intermediate in multi‐electron CO_2_ reduction on Cu surfaces [11]. Notably, under increasingly negative potentials, additional bands emerge at ∼1080 and ∼1120 cm^−1^, assignable to *CHO and *OCH_3_, which are critical intermediates in the CH_3_OH formation pathway. Similarly, in situ Raman spectroscopy of Sn‐coupled defective CuO catalysts clearly highlights the interaction between Cu active sites and surface‐bound intermediates (Figure 12b) [38]. Distinct peaks at ∼298 and ∼2083 cm^−1^ can be attributed to the frustrated rotational mode and C≡O stretching of adsorbed *CO, respectively. Additionally, a broad feature near ∼608 cm^−1^ corresponds to CuO_x_ species, suggesting the presence of low‐valent Cu states during CO_2_ reduction conditions favorable for CH_3_OH formation [127, 128]. These spectroscopic insights confirm the pivotal role of *CO and its downstream intermediates, such as *CHO and *OCH_3_, in tuning ECO_2_R toward CH_3_OH on Cu‐based catalysts. To further track the dynamic evolution of surface‐bound species and validate these intermediates under electrochemical conditions, complementary insights can be obtained from infrared spectroscopy techniques such as FTIR and SEIRAS.

### 4.2 Infrared Spectroscopy (FTIR and SEIRAS)

Infrared spectroscopy techniques, including FTIR and SEIRAS, are widely employed to detect surface‐bound intermediates and elucidate reaction pathways in ECO_2_R on Cu‐based catalysts, enabling time‐resolved monitoring of functional group transformations under realistic electrochemical conditions [129, 130, 131, 132]. During ECO_2_R, in situ FTIR study on CuS displayed bands at ∼ 1357 and ∼ 1448 cm^−1^, corresponding to *OCHO species, indicating a formate pathway with limited activity toward CH_3_OH due to the absence of further hydrogenated intermediates (Figure 12c) [103, 124]. On the other hand, CuV_0.1_S exhibited stronger CO_2_ adsorption (band at ∼ 2340 cm^−1^) and water binding (H─O─H bending at 1650 cm^−1^) due to V incorporation and the presence of higher‐valent Cu^+^/Cu^2+^ species (Figure 12d). Most importantly, bands at ∼1225 cm^−1^ (*CHO), ∼ 1390 cm^−1^ (*OCH_3_), and ∼ 1835 cm^−1^ (*CO‐bridge) were observed, confirming the formation of key intermediates for CH_3_OH production [133, 134]. This result highlights the role of V doping in enhancing *CO hydrogenation steps by modulating the electronic structure and active site environment (Figure 12e). In situ FTIR analysis on CuGa_2_ and Cu_9_Ga_4_ ordered intermetallic catalysts during ECO_2_R in 0.5 M KHCO_3_ revealed major bands at ∼ 1519/1373 cm^−1^ and ∼ 1520/1386 cm^−1^, respectively, corresponding to asymmetric and symmetric stretching of the OCO group in *HCOO–, indicating the formation of HCOO─ species via an O‐bound intermediate pathway [36, 135]. Isotopic ^13^CO_2_ labeling confirmed these assignments through downshifts to ∼ 1480 and ∼ 1333 cm^−1^ [136, 137]. The weak signals at 1808 and 2006 cm^−1^ for *CO‐bridge and *CO‐atop suggest minimal CO formation, supporting the favorability of the *HCOO route (path 2) over *CO‐mediated (*path 1*) CH_3_OH production as mentioned in the mechanism section. Additionally, peaks at ∼ 1225 and ∼ 1250 cm^−1^ indicate the presence of bidentate *HCOO‐ads and O─C─H bending, reinforcing the dominance of the HCOO‐mediated mechanism. The results establish that Ga incorporation modulates CO_2_ binding and facilitates selective HCOO^−^ accumulation, steering the reaction toward CH_3_OH via an oxygen‐bound intermediate pathway. In situ FTIR spectroscopy provided definitive spectral evidence for the formation and evolution of crucial adsorbed intermediates, including *CO, *CHO, and *CH_3_O, under ECO_2_R. The potential‐dependent appearance and transformation of these species indicate a stepwise hydrogenation pathway, strongly supporting the mechanistic route toward CH_3_OH formation. These findings highlight the key role of surface‐bound intermediates and their stabilization on Cu‐based active sites, providing valuable mechanistic insight into enhancing CH_3_OH selectivity in ECO_2_R.

### 4.3 X‐Ray Absorption Spectroscopy (XAS)

X‐ray absorption spectroscopy (XAS), which includes both X‐ray Absorption Near Edge Structure (XANES) and Extended X‐ray Absorption Fine Structure (EXAFS), serves as a powerful technique to investigate the electronic structure and local atomic environment of catalyst materials, especially under operando conditions [138, 139]. XANES provides very important information about the oxidation state and electronic configuration of metal centers, enabling real‐time monitoring of dynamic redox changes during ECO_2_R. On the other hand, EXAFS offers information about the local coordination geometry, bond distances, and structural dynamics around the active sites. Together, these techniques allow a comprehensive understanding of the evolution of catalyst structure and oxidation states during ECO_2_R, which is essential for determining the true active site of the catalyst. In one of the previous studies, XANES and EXAFS analysis revealed that the Cu_2_NCN catalyst possesses an electron state distinct from CuO and Cu_2_O, facilitating selective CH_3_OH formation. The Cu K‐edge 1s→4p transition peak of Cu_2_NCN (8983.0 eV) closely matches Cu_2_O (8982.1 eV), indicating a Cu(I)‐like valence state, but differs significantly from CuO (8988.2 eV), suggesting reduced Cu centers are preferable for CO_2_ activation (Figure 12f) [32]. EXAFS analysis revealed a single Cu–N coordination at ∼ 1.82 Å with a low coordination number (∼1.8), and long‐range Cu–Cu interactions were observed at R > 4.0 Å, implying significant electron delocalization (Figure 12g). These structural features, undercoordination, isolated Cu sites, and electron cloud delocalization due to NCN^2−^ ligand binding, create an optimal environment for stabilizing O‐bound intermediates (HCOO) and lowering the barrier for further hydrogenation, thereby promoting the CH_3_OH‐selective pathway in ECO_2_R [140]. Operando XAS study on CH_3_OH selective Sn‐coupled defective CuO (Sn_1_/V_o_–CuO–90) catalyst for both Sn and Cu K‐edges was also reported previously [38]. The Sn K‐edge XANES spectra revealed negligible changes in the Sn oxidation state during ECO_2_R, indicating the high stability of atomically dispersed Sn sites. In contrast, significant structural and electronic transformations were observed at the Cu sites during applied cathodic potentials (Figure 12h). Cu K‐edge XANES spectra depicted a progressive reduction of Cu species from +2 to 0 oxidation state as the potential was increased from −1.6 to −2.0 V vs Ag/Ag^+^. Linear combination fitting using CuO and Cu foil as references quantified the formation of Cu^0^, with its fraction rising from 15% at −1.6 V to 35% at – 2.0 V [141, 142]. Corresponding Fourier Transform (FT)‐EXAFS analysis revealed a notable decrease in the Cu─O coordination peak (∼ 1.5 Å) and an emerging Cu─Cu coordination peak (∼ 2.2 Å), consistent with the gradual reduction and restructuring of the CuO into metallic Cu (Figure 12i), which is believed to be the active phase facilitating CO_2_‐to‐CH_3_OH selectivity. Operando XAS analysis on Cu‐Ga‐based intermetallic catalyst revealed that the local coordination environment of Cu remained relatively stable during ECO_2_R, with only a minor evolution of Cu–O interactions and a slight shift in absorption edge, suggesting Cu retains a consistent metallic phase throughout the process [36]. In contrast, Ga sites exhibited significant changes under increasing negative potential, with a clear reduction of Ga^3+^ to metallic Ga. These results suggest that while Cu serves as a structural scaffold, the dynamic redox behavior of Ga, particularly the presence of surface gallium oxide, plays a crucial role in promoting CH_3_OH selectivity at lower potentials, which diminishes as Ga gets reduced at higher potentials. Operando XAS enables direct correlation between structural changes and catalytic activity by revealing oxidation states and local coordination of Cu‐based active sites during ECO_2_R to CH_3_OH. Future advancements in time‐resolved XAS at an industrial level current density in a flow cell or MEA configuration, will further enhance our understanding of reaction mechanisms, guiding the rational design of highly selective CH_3_OH‐producing catalysts [143].

## 5 Stability and Deactivation Mechanisms in Copper Catalysts

Several attempts have been made in the recent past to understand the stability of Cu‐based catalysts and their effective role in activity, selectivity, including the stability of the overall electrolysis. For industrial‐scale ECO_2_R applications, it is essential for Cu catalysts to deliver high performance, in terms of high current density and FE toward CH_3_OH, rather than producing a broad distribution of products. However, while significant efforts have been devoted to improving selectivity, comparatively less attention has been paid to the structural stability of these catalysts. The extensive degradation of the Cu catalysts during the ECO_2_R remains a major challenge, negatively impacting long‐term performance [144]. H. Wu et al. provided a thorough analysis of Cu‐based catalyst deactivation during the ECO_2_R [145] with generalized deactivation modes ranging from catalyst particle mobility to catalyst depletion. It was highlighted that although certain deactivation mechanisms, like segregation, coalescence, drastically impact the initial ECO_2_R selectivity, other substantial impacts could arise from salt precipitation and self‐inhibition of the catalyst materials. Predominantly, it can be understood that deactivation caused by oxidation state change during the reduction of CO_2_ shall significantly affect the overall ECO_2_R process. Prior studies depicted that defect formation induces a change in the crystal lattice, and those defects are precisely termed as active sites. For instance, oxide‐derived Cu (OD‐Cu) [146, 147] has early active sites at the onset potential, aiding in high conversion of CO_2,_ followed by a more thermodynamically stable phase if not stabilized initially, eventually leading to poorer performance and no longevity. Numerous reports demonstrated that OD‐Cu undergoes complete reduction immediately at the relevant onset potentials, although hydrocarbon (carbon product selectivity) selectivity is largely dependent on the stable OD‐Cu, resulting in enhancing C‐C coupling [148, 149]. It is obvious, as demonstrated in several previously reported literature, that OD‐Cu deliver enhanced ECO_2_R selectivity than a polycrystalline Cu, mainly due to the in situ generated intermediate active sites of OD‐Cu [150, 151, 152]. Alongside the defective species, other parameters like fragmentation and dissolution of the catalyst layers also lead to the deactivation of Cu, leading to a suppressed ECO_2_R process [153, 154].

Although the degradation mechanisms and deactivation of Cu catalysts have been extensively studied for the production of CO_2_ to high‐value products like C_2_H_4_ and C_2_H_5_OH, the understanding of degradation pathways under CH_3_OH‐selective conditions is still limited and remains an open question. Again, several recent studies demonstrate the conversion of ECO_2_R to CH_3_OH with Cu‐based catalysts, often limitations like low conversions or selectivity, low efficiencies and the stability of Cu limit the overall credibility ECO_2_R process in reaching the desired industrial level operations [144]. Before highlighting the deactivation of Cu, it is certainly vital to understand the mechanism of Cu deactivation by using techniques like operando/in situ XAS, monitoring the real‐time modifications of the Cu oxidation states. H. Kim et al. recently developed Cu and phosphate‐based hybrid catalysts Cu/Cu_2_P_2_O_7,_ reportedly for selective CH_3_OH synthesis [144]. With the support of in situ XAS, it was revealed that CP‐0.8 delivered a superior performance over the pristine CP throughout the ECO_2_R by maintaining the oxidation state of Cu, thereby highlighting the necessity to fabricate the OD‐Cu‐based hybrid materials. However, Bagchi et al. demonstrated that the Ga‐site is the active site by maintaining the coordination of Cu in their reported intermetallic catalyst Cu‐Ga_2_ based on in situ XAS studies [36]. Reportedly, Ga was subjected to leaching at elevated potentials from Cu‐Ga, highlighting the depreciation of overall CH_3_OH selectivity. These studies further delve into understanding the importance of co‐catalysts aimed at protecting the surrounding coordination of Cu and further highlighting the importance of maintaining highly selective yet stable catalyst systems. Current research on ECO_2_R to CH_3_OH is still limited to lab‐scale, with the current best‐performing catalysts often suffering from poor selectivity at higher currents, leading to deactivation. Therefore, while being on the path to attain higher selectivity coupled with long‐term operations, it is vital to develop Cu‐based catalyst systems that are immune to deactivation during the ECO_2_R.

## 6 Influence of Operational Conditions and Reactor Design

Although several d‐block metal complexes and alloys have been extensively investigated to tune selectivity toward hydrocarbons, Cu remains the benchmark catalyst due to its unique ability to selectively convert CO_2_ to hydrocarbons, including CH_3_OH, by effectively optimizing the binding energies of the reaction intermediates as elaborately discussed previously. However, even with an ideal electrocatalyst, other crucial process parameters, such as electrode potential, temperature, and pressure, as well as operating conditions like electrolyzer design and electrolyte selection, must be carefully optimized to achieve effective and efficient CH_3_OH conversion. In the following sections, we provide an overview of these parameters and provide key insights.

### 6.1 Electrode Potential

Due to the inherently sluggish kinetics of ECO_2_R, the reduction potential plays a critical role in overcoming kinetic barriers. Attaining a lower onset potential than the standard reduction potential (0.03V vs RHE) is essential to minimize electrolysis potentials and enhance the overall efficiency of the ECO_2_R. It is evident that the onset potential initiates the CO_2_ reduction at the interface by overcoming the specific activation barriers. By constantly varying the applied potential, selectivity shall be majorly altered through the modified reaction intermediates. However, at higher cathodic potentials directly elevate the partial currents at which Cu can fully be modified, thereby altering the intermediate and possible active sites. To avoid this, the incorporation of pulsed electrolysis can substantially aid in retaining the active site of Cu during long‐term operations. Furthermore, electrode potential can also be significantly controlled by engineering the electrode surface by replacing the conventional carbon‐based GDE with a polytetrafluoroethylene (PTFE)‐based GDE, followed by effective modulation of the surface microenvironment [155]. Although electrode potential is largely determined by the number of active sites, it is also noteworthy that electrolyte pH can substantially influence electrolysis potential during the ECO_2_R. It has been found in the previous study that tuning the electrolyte composition significantly impacts ECO_2_R efficiency. It was reported that Cu_88_Sn_6_Pb_6_ alloy structure altered product selectivity and rate of CH_3_OH formation by a mere tuning of the electrolyte composition, which in turn was achieved at a low applied potential vs Ag/AgCl [156]. In another study involving Cu(HHTQ) [144], a Cu‐based covalent organic framework (COF), precisely containing Cu^2+^ sites, attained a FE of 53.6 % at a low potential of −0.40 V_RHE,_ depicting the importance of lower electrolysis potentials in attaining a high selectivity toward CH_3_OH. These findings highlight that improving CH_3_OH selectivity in ECO_2_R is closely linked to operating at lower electrolysis potentials, thereby highlighting the necessity to design highly robust and efficient electrocatalysts.

### 6.2 Temperature and Pressure

It is well established that the overall process of ECO_2_R reaction rates can be altered by controlling the temperature, by affecting the thermodynamic equilibrium [157, 158]. Kim. et al., demonstrated at a current density of − 17–23 mA·cm^−2^ and in the presence of 0.5 M KHCO_3_, the CO_2_ reduction rate was higher at 0°C (3.06 10^−4^ molm^−2^s^−1^) than at 22°C (2.22 10^−4^ molm^−2^s^−1^) on Cu foil electrodes by lowering the competitive HER depicting the influence of temperature on ECO_2_R selectivity [159]. By lowering the temperature, CO_2_ solubility was enhanced further, resulting in a change of electrolyte pH change allowing the enhancement of the reaction ability and desired product distribution. Furthermore, it was also demonstrated that temperature changes can elevate the operating current densities [160, 161]. In another study, Pt/C‐based MEA electrodes [162] delivered a significant enhancement in CH_3_OH while working under lowered temperatures. It is understandable that the selectivity of ECO_2_R to CH_3_OH can be tailored by lowering the temperature, resulting in enhanced CH_3_OH production. Furthermore, it is also important to consider the pressure affecting the overall ECO_2_R process. By elevating the pressure, current densities can be elevated as well, depending on the type of electrolyzers used, which can be generally noticed in H‐type cells, where dissolved CO_2_ is available within the electrolyte. The pressurized CO_2_ speeds up the reaction and can modify product selectivity by enhancing the current densities [163, 164]. Previous reports, however, mentioned the disadvantages of high pressures, often being risky and increasing operational costs, making it a less explored parameter within ECO_2_R [165].

### 6.3 Electrolyzer Design and Configuration

**FIGURE 13:**
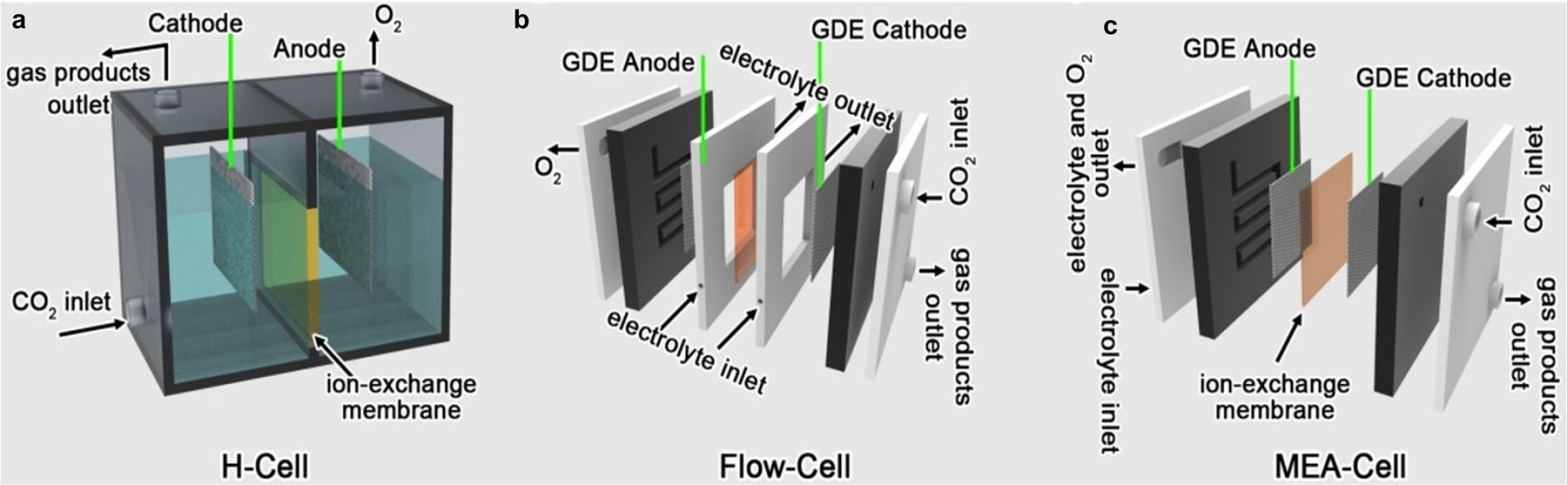
Types of electrolyzer configurations (a) Cell view for an H‐cell, (b) Cell view for a catalyst deposited on a gas‐diffusion layer (GDL) with a flowing catholyte channel, (c) cell view for a catalyst deposited on a gas‐diffusion layer with a non‐flowing catholyte (MEA) [169]. Reproduced with permission from [169]. Copyright 2022, Wiley‐VCH.

Extensive literature highlights the use of various electrolyzer configurations for the ECO_2_R process, ranging from traditional H‐cell configurations to more advanced flow cells and MEAs. Nevertheless, there is no standardized electrolyzer design to specifically tune CH_3_OH selectivity [166]. While two‐compartment‐based electrolyzers are commonly used, flow electrolyzers with separated cathode and anode chambers, separated by an electrolyte or ion‐exchange membrane, are widely adopted [167]. Many early studies primarily used H‐type cells, where the dissolved CO_2_ was selectively reduced to targeted products. Over recent years, investigations have been expanded further toward flow systems by deploying advanced porous GDE, which allows a continuous supply of CO_2_ and improves mass transport by enabling CO_2_ to diffuse through the microporous layer to the catalyst surface [167, 168]. This setup mitigates CO_2_ mass‐transfer limitation and suppresses the competing HER. More recently, MEA configurations, where the catalyst material is directly integrated with the ion‐exchange membrane, have been explored to minimize the distance between operating electrodes and advance scalability [169]. Figure 13 depicts the various electrolyzer configurations intended for ECO_2_R processes.

Recently, a MEA composed of a single‐atom Cu‐embedded porous carbon matrix [34] was reported, enabling CH_3_OH FE of over 44% at a current density of − 93 mA·cm^−2^ while maintaining the stability exceeding 50 h. While there is no significant reported literature demonstrating the importance of specialized electrolyzer design in governing the CH_3_OH selectivity, often with a well‐suited electrolyzer configuration, the overall improvements in parameters like activity, selectivity and single‐pass conversion can be greatly tuned for a superior overall ECO_2_R to CH_3_OH process. Some of the key points to be considered while designing the electrolyzer for enhanced selectivity are

1. Flow‐cell architecture, MEA and Zero‐gap assemblies, aided with a hydrophobic Gas Diffusion Electrode setup up aid in optimizing the CO_2_ mass transport while monitoring the active intermediates at the triple‐phase interface.
2. Altering the mass flow rate alongside regulating the partial pressure of CO_2_ over Cu‐based catalyst surfaces shall eventually aid in enhancing the CO_2_ to CH_3_OH conversion [144].
3. Prior experimental and computational studies revealed that managing the local concentration and modulating the pH of electrolyte for longer durations can significantly boost the overall CH_3_OH selectivity [144].
4. By curating the flow fields in MEA/ Zero‐gap assemblies, further delivery of CO_2_ is feasible, thereby regulating the optimized local availability.
5. Alongside the CO_2_ flow, electrolyte flow rates coupled with superior ion‐conductive membranes play a pivotal role in further improvement in CH_3_OH selectivity.
6. A tandem electrolyzer configuration can also be deployed where CO_2_ is initially converted to CO in the first electrolyzer while CO shall further be reduced to CH_3_OH in the latter connected in series. While these have already been demonstrated for hydrocarbons like C_2_H_4_ and C_2_H_5_OH [170, 171], resulting in a superior performance, it would be noteworthy to attempt the tandem mechanistic pathway, ensuring the CO reduction through the formate pathway, enhancing the CH_3_OH selectivity.

It can be summarized that by careful tuning of the electrolyzer configuration and tailoring the flow fields and flow rates, the overall ECO_2_R to CH_3_OH can be attained and shall further be directed to real‐time industrial scale operations.

### 6.4 Electrolyte Effects

Electrolyte medium can strongly influence the overall ECO_2_R mechanism, significantly affecting product selectivity and current density [172]. For CH_3_OH production, aqueous electrolytes are commonly used where water molecules act as proton sources for the reduction of intermediate species [173]. Additionally, the nature and size of the electrolyte cations can influence the intermediate adsorption on the catalyst surface, leading to significant changes in the reaction kinetics [95]. Apart from the conventional aqueous and organic electrolytes, more recent developments demonstrated the use of ionic liquids (ILs) [56, 57], which are proven to be advantageous by lowering the energy required to generate the reaction intermediates [174]. To tune the selectivity toward CH_3_OH, it is important to fabricate less viscous ILs, enabling high absorption of CO_2_. Traditional research conducted in H‐cells often deploys near‐neutral pH‐based electrolytes like KHCO_3_ and NaHCO_3_. Although bicarbonate‐based neutral electrolytes aid in minimal loss of CO_2,_ unlike the conversion to carbonates under alkaline conditions, these are often affected by lower conductivities. Most recent studies on Cu/Cu_2_P_2_O_7_ [144] depict that the pH of the electrolyte can profoundly influence the selectivity toward CH_3_OH while minimizing the competitive side‐reactions. A high FE of over 50% was reported under H‐cell conditions with KHCO_3,_ and a surprising 70.1% was attained when replaced with CsHCO_3,_ further highlighting the importance of size cationic species in the desired electrolyte. It is well understood that the choice of cations like K^+^ and Cs^+^ can substantially modulate the overall ECO_2_R process by stabilizing the key reaction intermediates adsorbed at the interface when compared with other further smaller cationic species like Li^+^ and Na^+^. While there are further reports demonstrating the use of varying electrolytes based on non‐Cu‐containing catalysts, it will be interesting to further focus on developing strategies electrolytes with varying compositions for Cu‐based materials.

### 6.5 Ion Exchange Membranes

**FIGURE 14:**
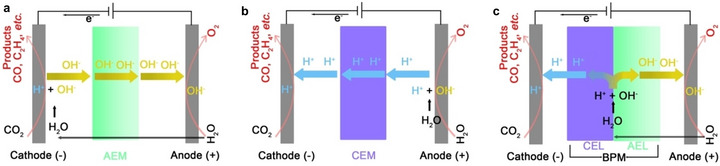
Schematic illustrations of the different types of ion transport through an A) AEM, B) CEM, and C) BPM for ECO_2_R. Reproduced with permission from [169]. Copyright 2022, Wiley‐VCH.

Along with catalysts, electrolyzers, and electrolytes, ion exchange membranes play a substantial role in tuning the selectivity toward CH_3_OH. While initial studies focused on cationic/proton exchange membranes, these often promoted HER. In this regard, anion exchange membranes and bipolar membranes have emerged as promising alternatives, demonstrating significant enhancement in CH_3_OH selectivity and overall activity [175, 176]. Figure 14 illustrates the various ion exchange membranes intended for the ECO_2_R process and their associated ion transport mechanism. Overall, ECO_2_R to CH_3_OH performance is greatly influenced by membranes mainly through three integrated mechanisms. The obvious cation and anion migration is controlled by the modulated electrode's surface interface, influencing the reaction pathways, local surface microenvironment, adjusting the localized pH and reactant concentration, followed by ion management and product adsorption on the surface, further preventing the unsought product crossover. Alongside the ion‐exchange membranes, ionomer layers can directly influence the surface microenvironment, thereby modulating the localized CO_2_/H_2_O ratio. Nevertheless, for a superior ECO_2_R to CH_3_OH, a bipolar membrane [176] with an appropriate thickness, water uptake capability with optimized ionic mobility shall govern in attaining elevated high Faradaic efficiency.

## 7 Challenges in Scaling Up Methanol Production

ECO_2_R is often considered to be less efficient in delivering highly selective products at marginalized costs when compared to the conventional thermal hydrogenation processes, which are already industrially implemented. CH_3_OH is mainly synthesized from syngas produced by steam reforming of CH_4_ at huge industrial levels to manage the global supply chain [177]. Nevertheless, despite its sustainability potential, the ECO_2_R to CH_3_OH process is extremely challenging both in terms of environmental and technological aspects due to the low energy efficiency and poor product selectivity. Key hurdles include the fabrication of electrocatalysts that are both long‐term stable and selective at economically viable conditions. These catalysts should operate at low electrolysis potentials while minimizing the highly competitive side reaction of HER, thereby elevating the energy efficiency of the system. Cell potentials are to be kept under a bare minimum of 3–3.5 V to achieve an overall energy efficiency of at least 50%. The present mechanistic understanding of Cu‐based catalyst degradation during CH_3_OH‐selectivity via operando approaches remains unclear. Therefore, gaining deeper insight through operando and in situ characterization, supported by theoretical studies, is of utmost importance. Furthermore, selectively stabilizing the reaction intermediates like *CO and *HCHO (formate pathway) remains a challenging task as *CO can desorb as CO or further dimerize to C_2+_ products, leaving lower selectivity toward CH_3_OH. As stated previously, the structural deactivation and reconstruction of Cu must be optimized to stabilize the overall ECO_2_R process while maintaining the desired CH_3_OH selectivity while operating at improved energy efficiency. To improve energy efficiency, ECO_2_R to CH_3_OH selectivity can be elevated by combining with the conventional thermocatalytic process and even using tandem systems. To successfully implement the different coupling techniques involved on the industrial scale, it is obvious that further research and development are highly recommended and essential.

## 8 Emerging Opportunity of Cu‐Catalyzed ECO_2_R for Methanol Production Compared to Industrial Thermocatalysis

The ECO_2_R presents a fundamentally unique and increasingly promising technology compared to the conventional thermochemical process (TCO_2_R), especially when evaluated with respect to catalyst tunability, energy integration, and system flexibility [178, 179]. Traditional TCO_2_R processes, such as Cu/ZnO/Al_2_O_3_‐based catalytic hydrogenation of CO_2_ for CH_3_OH synthesis, mainly rely on elevated temperatures (>200°C) and high H_2_ pressures (60‐100 bar), which constrain catalyst design and induce issues like coking, sintering, and limited regeneration [180]. In contrast, ECO_2_R occurs under milder conditions and demonstrates the high surface area and compositional tunability of Cu‐based nanostructures, enabling control over local reaction environments and intermediate stabilization as discussed in the previous sections. While both catalytic processes display sensitivity to the oxidation state of Cu and dopant effects, the ability to precisely tune the electronic structure, local coordination, and morphology under operando conditions gives ECO_2_R a unique advantage in controlling selectivity toward CH_3_OH [102, 181, 182]. Furthermore, the compositional strategies such as dual‐doping or support modulation, widely explored in thermocatalysis, can also be translated into electrochemical processes in the Cu‐based catalyst system due to the lower thermal constraints and modular reactor designs [183]. From a systems point of view, ECO_2_R can easily be coupled with intermittent renewable power, offering real‐time control over reaction rates and facilitating CH_3_OH synthesis directly from CO_2_‐rich streams [184]. Despite its lower current technology readiness, the ECO_2_R minimizes fossil energy reliance, enhances carbon circularity, and opens a tunable, sustainable pathway for CH_3_OH production over thermocatalytic capabilities.

## 9 Perspectives and Future Directions

**FIGURE 15:**
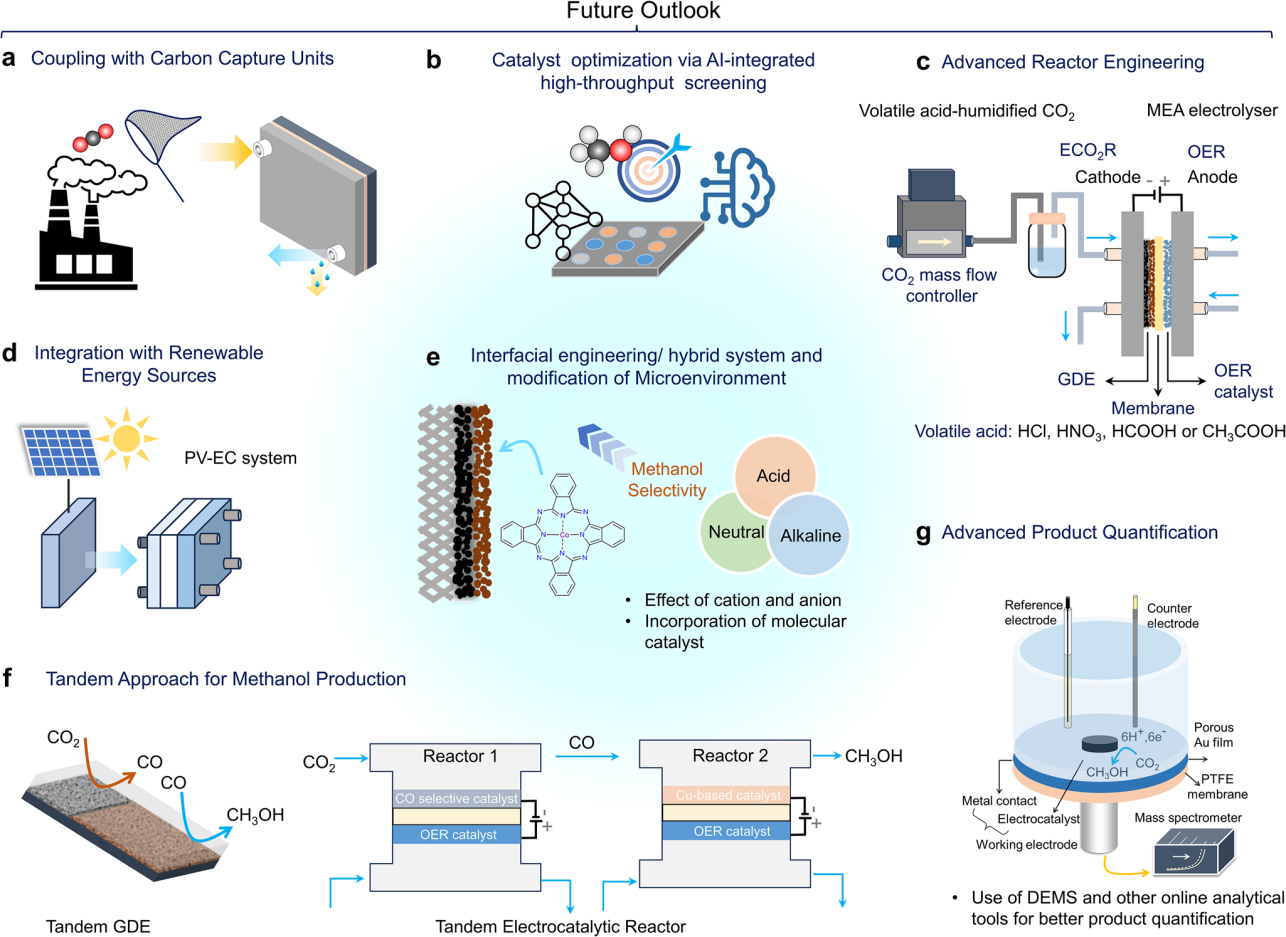
Future perspective for ECO_2_R to CH_3_OH using Cu‐based catalysts in, highlighting (a) coupling with carbon capture units, (b) AI‐driven high‐throughput screening of various catalyst materials, (c) innovative reactor designs [186], (d) integration with renewable energy sources, (e) engineering of interfacial and microenvironment of electrode‐electrolyte interface [187], (f) tandem electrode/reactor systems [188, 189] and (g) the incorporation of advanced product quantification techniques (e.g., differential electrochemical mass spectrometry, (DEMS)).

Despite the significant promise of ECO_2_R toward mitigating CO_2_ emissions and producing value‐added chemicals toward a net‐zero carbon future, substantial efforts are still required to close the CO_2_ cycle. Accelerated current research at the lab scale must be translated into higher technology readiness levels (TRL), aiming at immediate industrial‐scale applications. Simultaneously, progress in CO_2_ capture technologies should be prioritized, and further focus should be dedicated to integrated systems involving capture and conversion (Figure 15a). The present section of the review presents our perspectives on the current state of the field while outlining the immediate future directions for establishing a more sustainable and scalable ECO_2_R to CH_3_OH process.

### 9.1 Rational Design of Catalysts and Electrochemical Reactor Development for Improved CH_3_OH Selectivity

In the previous sections, significant emphasis was given to ongoing developments in the design and fabrication of Cu‐based electrocatalysts. While high FEs are often reported, the overall ECO_2_R process still suffers from low current densities [36] and poor energy efficiencies. In this regard, it is crucial to rationally design electrocatalysts that are not only highly efficient and stable but also capable of selectively promoting CH_3_OH production. As the ECO_2_R to CH_3_OH process follows a 6 e^−^ transfer pathways involving *CO and/or *HCOO reaction intermediates, the ideal electrocatalyst should favor a thermodynamic reduction potential of at least 20 mV more positive than that of HER [93, 94, 95]. Among various transition metals, Cu has the ability to uniquely enable hydrocarbon formation from CO_2_; still, it exhibits inherently low selectivity for CH_3_OH [71]. Although pure Cu lacks the intrinsic ability to selectively produce CH_3_OH, tailored structures and surfaces of Cu [26, 46, 47, 48] and bimetallic or alloy structures often result in better CH_3_OH selectivity [36, 52, 53]. Hence, future efforts should be strategically focused on rational design of structurally complex yet operationally efficient electrocatalysts, particularly through the integration of Cu with additional metal components employing artificial intelligence (AI)‐driven predictive modeling in combination with high‐throughput screening methodologies, and autonomous robotic platforms for accelerated performance study (Figure 15b) [185]. Future development in advanced reactor and process engineering for highly selective CH_3_OH production at industrially relevant current density using MEA configurations may focus on integrating volatile acid vapors into the CO_2_ feed stream as a novel strategy to suppress cathodic salt accumulation, thereby enhancing system stability and enabling more efficient ECO_2_R performance (Figure 15c) [186].

### 9.2 Integration with Renewable Energy Systems for Sustainable CH_3_OH Production

Although the lab‐scale research usually employs commercially available potentiostats or power sources powered by conventional electricity, the long‐term vision is to operate ECO_2_R systems using intermittent stored renewable energy (solar energy). Real‐time exploration of solar‐driven electrochemical processes for CH_3_OH production represents a promising route toward sustainable energy solutions by producing high‐value‐added products ^47^. Currently, the solar‐driven ECO_2_R to CH_3_OH production remains at a low TRL (2‐3) as most systems remain at laboratory scale, with a challenge in low conversion efficiency and operational stability [190]. Ongoing progress in light‐absorbing materials and electrochemical technologies continues to improve the performance of decoupled PV‐EC (photovoltaic‐electrochemical) systems, helping to mitigate their higher costs. For practical integration of PV absorbers with electrochemical cells, a thorough understanding of their behavior under real operating conditions is essential [191]. The effective integration of the PV‐EC system highlights the importance of optimizing the geometric areas of both the light absorber and the electrochemical reaction components, thereby enhancing the overall system performance and increasing the solar‐to‐fuel (STF) efficiency (Figure 15d). Furthermore, the efforts to integrate solar‐driven electrochemical processes into larger energy systems and industrial applications are vital for the practical deployment and scaling of sustainable technologies. Such integration enables the use of abundant solar energy for CH_3_OH synthesis via ECO_2_R, providing a renewable alternative to fossil fuels. Achieving this vision requires interdisciplinary collaboration, starting from material science, electrochemistry, and renewable energy engineering to innovate device designs, improve efficiency, and ensure system stability under real‐world conditions. Additionally, these systems with industrial processes, such as wastewater treatment or chemical manufacturing, could further enhance resource efficiency and accelerate the transition toward a sustainable CH_3_OH economy and broader renewable energy adoption [192, 193].

### 9.3 Potential of Hybrid/Tandem Systems: Combining Cu with Other Catalytic Approaches

Cu alone has a lower selectivity to produce CH_3_OH, and therefore, Cu is often combined with other metals either as a bimetallic or intermetallic material, thereby enabling high CO_2_ to CH_3_OH conversion [36, 52]. For comparison, single‐step methods involving additional catalytic sites like Cu_1_._63_Se catalysts [35] achieve a FE of 77.6% for CH_3_OH at a current density of − 41.5 mA·cm^−2^, while Ag/S‐doped Cu_2_O/Cu reaches a FE of 67.4% at − 122.7 mA·cm^−2^ [37]. Future research may explore interfacial engineering of hybrid systems combining molecular cobalt complexes with Cu‐based catalysts, alongside electrolyte cation tuning, to synergistically enhance ECO_2_R to CH_3_OH selectivity by employing complementary catalytic pathways and optimized PCET dynamics (Figure 15e) [187, 194]. Moreover, tandem strategies for ECO_2_R to CH_3_OH involve multi‐step processes to enhance the FE and energy efficiency by optimizing intermediate steps and specialized catalysts (Figure 15f). Lately, an anodized titanium catalyst containing Ti^3^^+^ sites and oxygen vacancies (termed as TOVs) was discovered that efficiently reduces HCOO^−^ to CH_3_OH, achieving a FE of 12.6% at − 1.0 V vs RHE. This tandem approach first reduces CO_2_ to HCOO^−^, followed by further reduction of HCOO^−^ to CH_3_OH, thereby overcoming the adsorption‐energy scaling limitations that hinder direct ECO_2_R to CH_3_OH. The TOVs act as active sites facilitating a vacancy‐filling pathway mediated by *H_2_COOH intermediates, enhancing CH_3_OH production. This strategy provides guidelines for designing catalysts by optimizing *HCOOH and *H_2_COOH binding energies and vacancy formation energies, enabling more efficient CH_3_OH synthesis from tandem ECO_2_R [195]. In a 2025 report, a sun‐driven tandem strategy has been shown to improve CH_3_OH production from CO_2_ by first electrochemically converting CO_2_ into syngas (H_2_:CO ≈ 2) using a self‐supporting catalyst with Ni single atoms and encapsulated Co NPs, achieving over 90% FE for CO and stable syngas composition under solar‐powered conditions. The syngas is then fed into a photothermal reactor powered by sunlight, where it is converted into high‐purity CH_3_OH (>97% volume fraction) at a rate of 0.238 g CH_3_OH per gram catalyst per hour. This tandem approach integrates PV‐driven ECO_2_R and photothermal CO hydrogenation, enabling efficient solar energy storage in CH_3_OH with significantly higher purity and yield than direct electroreduction methods while operating under mild conditions and reducing the energy‐intensive purification step [196]. As mentioned previously, in the electrolyzer design concepts, potential tandem devices with electrolyzers connected in series mode and operating in a cascade mechanistic pathway would be one of the promising strategies to efficiently convert CO_2_ to CO and CO to CH_3_OH. Nonetheless, high single‐pass conversion of CO_2_ to CO shall not be compromised, and if neglected otherwise, unreacted CO_2_ at the second CO to CH_3_OH electrolyzer could suffer from lower selectivity and stability. To circumvent this, intermediate CO_2_ absorption chamber can be installed ensuring the elimination of unreacted CO_2_ and further enhancing the CO to CH_3_OH conversion.

### 9.4 Opportunities for In‐Depth Mechanistic Studies and Precise Product Quantification Using Advanced In Situ Tools

There has been an increasing trend in the development and implementation of in‐situ and operando techniques to characterize the evolution of electrocatalysts, and there is further scope for adopting more sophisticated and even hybrid analytical methods. Such tools are essential to gain deeper insights into the surface of electrocatalysts, compositional and morphological changes, along with the formation of critical reaction intermediates. Advanced in‐situ tools offer significant opportunities for mechanistic studies of ECO_2_R to CH_3_OH by enabling real‐time observation of catalyst structure, reaction intermediates, and surface dynamics under operating conditions. While surface morphology evolution and catalyst reconstruction can be studied by using in‐situ/operando atomic force microscopy (AFM) and TEM techniques to find out the origin of the CH_3_OH selectivity, reaction intermediates and products can be traced by implanting DEMS (Figure 15g) [197, 198, 199, 200]. Future work should focus on the development of time‐resolved, multi‐modal operando techniques, integrated with emerging tools such as machine learning, automated high‐throughput screening, and advanced Spectro‐electrochemical diagnostics, to enable precise correlation between dynamic structural transformations, reaction intermediates, and methanol selectivity on Cu‐based catalysts.

### 9.5 Techno‐Economic Analysis and Outlook for Industrial Scale Set‐Up

Moving forward, ECO_2_R presents a compelling strategy for sustainable CH_3_OH production by enabling direct CO_2_ conversion under ambient conditions, with the potential to integrate seamlessly with intermittent renewable energy sources and replace energy‐intensive multistep processes. Increasing governmental interest is evident through emerging policies aimed at promoting CO_2_ utilization in combination with renewable energy integration. To understand the economic complications of further scale‐up, there are several factors to be considered with the aim of making the overall ECO_2_R to CH_3_OH a viable alternative. Major critical considerations pertaining to ECO_2_R to CH_3_OH specifically include the costs of production, carbon footprint, followed by the demand and price of CH_3_OH in the global market. At the implementation level, factors like large‐scale electrolyzer design comprising batch or stack configurations, handling the end products and often ease of product separation play a dominant role in translating this from lab to industrial scale operations. For example, alkaline flow cells have higher energy efficiency but suffer from high CO_2_ loss due to carbonate formation, further increasing regeneration costs. However, MEA electrolyzers operating under near‐neutral pH exhibit higher cell voltages but substantially lower carbonate‐related costs, making them currently a more economically viable alternative [201].

- **Cost Reduction Strategies**: Recent studies concerning the techno economic analysis (TEA) demonstrate that the levelized market costs for ECO_2_R to CH_3_OH can be as low as $190 per ton when compared to natural gas based production costs of $100 to 200 and a surprising higher production costs for bio CH_3_OH generated from bio‐gasification plants ranging over $ 370–760 per unit ton, further depicting the economic viability of electrochemical CH_3_OH production [202]. One such strategy for initial reduction of costs is to reduce carbonate formation and crossover. Improving the energy efficiency (>70% for alkaline cells, >50% for MEA), followed by lowering electricity costs, is crucial for keeping CH_3_OH production costs below $400/ton, thereby making the process potentially profitable [201]. However, these prices largely depend on the local raw material and electricity costs. Although significant cost‐effective strategies can be attained by achieving the highest FE % at minimal cell potentials, it is extremely vital to replace the existing use of energy‐driven electrolysis to further lower the costs of operations.
- **Operational parameters**: Key significance must be attributed to the operational parameters like current density, operational stability, scalability factors and attaining the maximum FE %. Currently, the best performing ECO_2_R to CH_3_OH electrolyzers operate at current densities ranging from ∼ − 50–100 mA·cm^−2^, which need to be increased to a target of operating at − 500–600 mA·cm^−2^ while maintaining a low cell potential strictly limited to a mere 3V (E _cell_). Under these elevated operating parameters, it is still important to design the full‐scale electrolyzer operating for thousands of hours of continuous operation.
- **Global Market Outlook**: The global CH_3_OH market is projected to reach $26 billion by 2025 with steady growth. The gradual integration of renewable (green) CH_3_OH alongside fossil‐derived CH_3_OH is expected, supported by policy incentives to overcome current cost barriers, ensuring the long‐run economic capital gains via ECO_2_R [203].
- **Current Costs and Scale‐Up**: Building an ECO_2_R to CH_3_OH plant is estimated to cost around €20 million, with CH_3_OH production costs currently around €500‐600 per ton in the EU. These costs are higher than fossil‐based CH_3_OH but are expected to decrease significantly by 2050 due to cheaper renewable electricity and improved electrolyzer efficiency [203]. As depicted earlier, relatively cheaper market costs were feasible by thoroughly ensuring cost reduction strategies are met with the successful integration of renewable energy‐driven ECO_2_R to CH_3_OH; cost economics are still to be contemplated, as the market costs can greatly be affected by the concerned local demand and supply of critical raw materials.
- **Environmental Impact**: ECO_2_R‐to‐CH_3_OH conversion using renewable electricity offers significant greenhouse gas reductions, aligning with decarbonization goals and providing sustainable fuel and chemical feedstock alternatives, making it almost an environmentally safe strategy. ECO_2_R to methanol production complements renewable energy and green hydrogen efforts, advancing circular carbon goals through parallel sustainable routes [201]. Most importantly, while all the factors above are thoroughly monitored, carbon emissions from the overall ECO_2_R process should be under strict vigilance, therefore highlighting the supremely advantageous route of ECO_2_R to CH_3_OH over the conventional routes.

## 10 Conclusions

In this review, we provide a comprehensive overview of ECO_2_R to CH_3_OH, with a particular focus on Cu as the principal electrocatalyst. We highlight current research directions and associated challenges, and recent progress in the field, which has seen significant advancements over the past decade. Numerous Cu‐based modified electrocatalysts have been reported and evaluated across diverse electrolyzer configurations and operating conditions. Considering the complexity of this field, there is a pressing need for the scientific community to develop and critically assess advanced electrochemical systems. Here, we have critically examined the role of Cu in CH_3_OH production, categorizing various Cu‐based catalyst systems through a comprehensive state‐of‐the‐art literature survey. Additionally, we also explore proposed reaction mechanisms, identifying key intermediates, pathways for CO formation, and subsequent hydrogenation steps leading to CH_3_OH. Competing reactions and the influence of the local reaction environment are discussed, supported by theoretical insights from DFT. Special attention is given to in‐situ and operando characterization techniques, which are essential for elucidating active sites and mechanistic pathways. While notable advances have been made in understanding reaction kinetics and mechanisms, several unresolved questions remain, such as an in‐depth understanding of the actual active site and the complete reaction pathways, and the limited selectivity of catalysts under industrially relevant conditions. Similarly, we also address catalyst performance metrics, degradation protocols, and the impact of electrolyzer design and operating parameters. Despite there is growing interest in scaling up CH_3_OH production, barriers remain to achieving commercially viable systems. Finally, we emphasize the importance of rational catalyst design through electronic structure modulation to stabilize *CHO intermediates by tuning Cu‐O interactions. Optimization of the electrochemical reactor and the microenvironment is also crucial for enhancing methanol selectivity over competing products. Furthermore, integration with renewable energy systems and techno‐economic analysis is briefly discussed, concluding that there is a need for continued laboratory‐scale research to bridge the gap toward industrial CH_3_OH production.

## Conflicts of Interest

The authors declare no conflict of interest.
